# Effects of early water, sanitation, handwashing, and nutrition interventions on child development at school age: a follow-on study of a cluster-randomized trial in rural Bangladesh

**DOI:** 10.1371/journal.pmed.1004793

**Published:** 2025-12-16

**Authors:** Fahmida Tofail, Helen O. Pitchik, Mahfuza Islam, Rizwana Khan, Abul K. Shoab, Fahmida Akter, Shirina Aktar, Tarique M. N. Huda, Mahbubur Rahman, Peter J. Winch, Stephen P. Luby, Lia C. H. Fernald

**Affiliations:** 1 Nutrition Research Division, icddr, b, Dhaka, Bangladesh; 2 Division of Epidemiology, School of Public Health, University of California, Berkeley, Berkeley, California, United States of America; 3 Division of Environmental Health Sciences, School of Public Health, University of California, Berkeley, Berkeley, California, United States of America; 4 Enteric and Respiratory Infections, Infectious Disease Division, icddr, b, Dhaka, Bangladesh; 5 Environmental Health and WASH, Health System and Population Studies Division, icddr, b, Dhaka, Bangladesh; 6 Department of Health Promotion, Education, and Behavior, Arnold School of Public Health, University of South Carolina, Columbia, South Carolina, United States of America; 7 Department of Public Health, College of Applied Medical Sciences, Qassim University, Buraydah, Saudi Arabia; 8 Global Health and Migration Unit, Department of Women’s and Children’s Health, Uppsala University, Uppsala, Sweden; 9 Department of International Health, Johns Hopkins Bloomberg School of Public Health, Baltimore, Maryland, United States of America; 10 Division of Infectious Diseases and Geographic Medicine, Stanford University, Stanford, California, United States of America; 11 Division of Community Health Sciences, School of Public Health, University of California, Berkeley, Berkeley, California, United States of America; Washington University In St Louis: Washington University in St Louis, UNITED STATES OF AMERICA

## Abstract

**Background:**

A previous cluster-randomized controlled trial in Bangladesh found that individual or combined water, handwashing, sanitation, and nutrition interventions during pregnancy and after birth improved developmental outcomes of children at 1 and 2 years of age. In this study, we aimed to determine if these intervention effects were sustained for children at school age.

**Methods and findings:**

Clusters of pregnant women were enrolled between May 31, 2012 and July 7, 2013 and block-randomized into chlorinated drinking water (W); improved sanitation (S); handwashing with soap (H); combined WSH; nutrition counseling and provision of lipid-based supplements (N); combined WSH + N, or a double-sized passive control arm (C) with no intervention visits (*N* = 5,551). The primary outcomes of the main trial after the 2-year intervention were 7-day diarrhea prevalence and length-for-age *z*-score, measured in 4,584 children of enrolled pregnant women. We conducted a post hoc, follow-up of all initially enrolled mothers and their children 5 years after intervention completion, when children were 7 years old. Primary outcomes were child cognition assessed using the Wechsler Pre and Primary Scale of Intelligence (WPPSI-IV), along with assessments of fine motor abilities, behavior, school achievement, and executive function; secondary outcomes were maternal mental health and stimulation in the home environment. We conducted intention-to-treat analyses using generalized linear models to calculate unadjusted and adjusted comparisons between each arm and the control group, accounting for block-level clustering.

Between September 2019 and February 2021, we re-enrolled 4,175 households from all 720 original clusters, with the full set of child development assessments conducted on 3,833 children across 718 clusters. Children in the WSH + N, N, and S arms had improved cognitive scores on one or more domains compared to the control arm, with adjusted effect sizes between 0.10 (95%CI: 0.00, 0.20) and 0.15 (0.03, 0.27). Children in the W, H, N, WSH, and WSH + N arms demonstrated improved prosocial behaviors (adjusted effect sizes between 0.20 (0.07, 0.33) and 0.31 (0.16, 0.46)) and reduced difficult behaviors (adjusted effect sizes between −0.15 (−0.28, −0.01) and −0.31 (−0.45, −0.17)). No intervention effects were observed for fine motor, executive functioning, or school achievement outcomes. Maternal depressive symptoms were improved in the WSH + N, H, and N arms (adjusted effect sizes between −0.14 (−0.24, −0.03) and −0.21 (−0.31, −0.11)), and the stimulating home environment was improved in all intervention arms (adjusted effect sizes between 0.17 (0.01, 0.33) and 0.40 (0.25, 0.56)). Children whose families had higher wealth at baseline and those who were male tended to have larger effect sizes on the FSIQ. Data collection for this study was interrupted by a 6-month pause at the start of the COVID-19 pandemic. The main limitation of this study is loss to follow-up.

**Conclusions:**

At 7 years of age, we found small, sustained benefits of early water, sanitation, handwashing, and nutrition interventions on child cognitive and socioemotional outcomes, the stimulating home environment, and maternal mental health. Future work to determine the mechanisms underlying these intervention effects will further inform the design of early interventions to improve child health and development.

**Trial registration:** Follow-up trial: ClinicalTrials.gov, NCT04443855. Original WASH-Benefits Bangladesh (WASH-B): ClinicalTrials.gov, NCT01590095.

## Author summary

**Why was this study done?:**

- An earlier trial of water, sanitation, handwashing, and nutrition interventions had impacts on child development at 2 years of age.
- There is little evidence about whether these specific early interventions have continued impacts in middle-childhood.

**What did the researchers do and find?:**

- We conducted a follow-up assessment of 7-year-old children who had been enrolled in a cluster-randomized controlled trial of water, sanitation, handwashing, and nutrition interventions in early life.
- We found small, sustained benefits of these early interventions for child cognitive and socioemotional outcomes, the stimulating home environment, and maternal mental health.

**What do these findings mean?:**

- Water, sanitation, handwashing, and nutrition interventions in the first 2 years of life can have lasting effects on key outcomes for children at 7 years of age.
- The main limitation of this study is loss to follow-up; the children who had complete assessment represented 69% of the initially enrolled sample.

## Abstract 3

In this follow-up of a randomized controlled trial, Fahmida Tofail, Helen Pitchik and colleagues assess the long-term effects of early water, sanitation, handwashing and nutrition interventions on child development in rural Bangladesh.

## Introduction

In low- and middle-income countries (LMICs), one-third of 3 and 4-year-olds fail to meet basic milestones in cognitive or socioemotional development [1]. Early measures of motor, cognitive, and socioemotional development can be predictive of later life outcomes, including educational attainment, economic earnings, and socioemotional behaviors in early adulthood [2–4]. During early childhood when the brain undergoes rapid structural and functional changes, positive experiences are more likely to contribute to the development of synaptic connections important for optimal developmental trajectories and resilience, and negative experiences can shift a child off the optimal developmental trajectory [5]. Thus, interventions to reduce risk factors for impaired development in early life are critical for promoting positive developmental trajectories and later life outcomes.

Similar to the classification of nutrition interventions, interventions targeting child development can be classified as development-specific interventions (i.e., addressing immediate and direct determinants of child development, such as unsupportive caregiving practices), or development-sensitive interventions (i.e., addressing the underlying causes of poor development such as poverty, food insecurity, or poor water and sanitation) [6]. Development-specific interventions that teach caregivers about the developmental importance of responsive caregiver-child interactions, can impact immediate early child development outcomes [7].

Development-sensitive interventions include those that provide nutritional supplementation in early childhood, as well as those that aim to improve water, sanitation, and hygiene (WASH) conditions. Nutrition interventions, which provide the nutrients required for brain development can improve child development outcomes early in life when delivered in the first 1,000 days [8]. WASH interventions aim to reduce the burden of enteric pathogens in the environment. Enteric infections, including intestinal worms caused by poor sanitation, can result in iron deficiency, and have negative consequences for child development outcomes [9,10]. Observational studies have demonstrated associations between diarrhea or other infectious diseases and impaired cognitive outcomes [11]. Though observational research demonstrates associations between improved water and sanitation infrastructure and early child development outcomes [12], few randomized-controlled trials have evaluated the independent effects of early WASH interventions on child development outcomes. Three interventions, including the one reported here [13], have evaluated the immediate post-intervention effects of development-sensitive interventions targeting improvements in WASH in the first 2 years of life on child development outcomes [14,15]. An early WASH intervention in rural Zimbabwe found no effect on child development outcomes at 2 years of age [14]. Individual or combined WASH and nutrition interventions improved early child development at age two in Bangladesh, but not in Kenya [13,15].

Despite robust evidence for the early effects of development-specific interventions, very few studies have investigated the medium- or long-term impacts of early interventions to promote child development on outcomes in middle and late-childhood [16]. There is even less evidence for the medium- or long-term effects of development-sensitive interventions. A follow-up of the early WASH intervention in rural Zimbabwe that had no impact on development outcomes in early childhood found no impacts on cognitive development at 7 years of age, and small impacts of WASH on socioemotional function [17]. Another cluster-randomized controlled trial (cRCT) in Pakistan found that children whose households received a handwashing promotion and drinking water treatment for 9 months in the first 30 months of life had improved child development at 5–7 years of age [18]. A cRCT in rural India evaluated the impact of community-led total sanitation campaign (which increased sanitation coverage) on later cognitive outcomes found improvements in intelligence among 7–15 year-olds living in villages that had received the intervention 10 years previously [19]. Our team was unable to find any studies that evaluated the later impact of an early combined Nutrition and WASH intervention that had previously demonstrated an early impact on child development outcomes.

Our previous cRCT in Bangladesh evaluated the impact of water quality, handwashing, sanitation, and nutrition interventions, when delivered either individually or in combination from pregnancy through 18 months after birth, on diarrhea, growth, and developmental outcomes in children [13,20]. We hypothesized that the WASH and Nutrition interventions had the potential to positively affect the developmental trajectories of children by reducing enteric infection, improving child nutritional status and health, and altering parental interaction and care practices. At the immediate, post-intervention evaluation at the age of ~2-years, there were benefits on one or more domains of child development in all intervention arms, with the greatest benefit in the combined intervention arm (WASH and nutrition), and effect sizes ranging from 0.14 to 0.37 SDs [13]. Additionally, mothers in all intervention groups reported fewer depressive symptoms compared to mothers in the control group (effect sizes ranged from −0.19 to −0.29 SDs). In the current study, we aimed to evaluate the effects of individual and combined water, sanitation, handwashing, and nutrition interventions on primary outcomes of child development, school achievement, executive functioning, fine motor development, and socioemotional development, and secondary outcomes of maternal mental health and stimulation in the home environment when children were 7 years of age.

## Methods

### Ethics

The study protocol was approved by Ethical Review Committee at icddr,b (PR-19025), and the Committee for Protection of Human Subjects at the University of California, Berkeley (2018-12-11672). Formal written consent was given by caregivers for their participation as well as that of their child, and verbal assent was given by children who participated in the study.

### Study design and participants

The parent cluster-randomized controlled trial (WASH-Benefits, or WASH-B, ClinicalTrials.gov Identifier: NCT01590095) was conducted in the rural villages of four districts (Gazipur, Kishoreganj, Mymensingh, and Tangail) of central Bangladesh. The parent trial began in 2012 when there were no major water, sanitation, or focused nutrition programs in the study area. Initially enrolled participants were pregnant women, with their in-utero children considered the index children for the intervention contents and subsequent assessments.

### Randomization and masking

The details about randomization, study design, intervention, methods, and rationale are described elsewhere [20,21]. In brief, 720 clusters, each consisting of 6–8 eligible households (a pregnant woman who expected to deliver in the 6 months following the baseline survey lived in the household) were randomized. Block-randomization was applied within geographically proximate clusters, with block size of 8. In each of these blocks of 8 clusters, one cluster was randomized to each of six active intervention arms, and two to the passive control arm. Arms included: chlorinated drinking water (W); improved sanitation (S); handwashing with soap (H); combined water, sanitation, and handwashing (WSH); improved nutrition through counseling and provision of lipid-based nutrient supplements (N); and combined water, sanitation, handwashing, and nutrition (WSH + N) or a double-sized, passive, control arm (C) (Table A in S1 Text). The control arm did not include any intervention visits or contact with the intervention implementation team, and participants were not masked to intervention status. The intervention continued for 2 years. A total of 5,551 pregnant women were enrolled, among them, 4,757 live births were assessed for child development outcomes at 1 year and 4,403 at 2 years of age [13].

### Procedures

Between September 2019 and February 2021, 7 years following intervention initiation, and 5 years following intervention completion, we re-visited the households of all live births who had not been reported at any previous time point to have passed away. Trained enumerators evaluated children, mothers, and the home environment through two separate household visits around a week apart. The visits took 1.5 and 2.5 hours, respectively. During the initial visit (Day-1), trained enumerators obtained written consent from caregivers and collected information on sociodemographic factors as well as data on water, sanitation, and handwashing infrastructure. Additionally, they evaluated the home environment, children's behavior, and anthropometry. In the subsequent visit (Day-2), enumerators obtained additional verbal assent from children and assessed the developmental outcomes of the children and evaluated the depressive symptoms of their mothers.

Day-1 enumerators were 10 university graduates who completed a 2 week training. Day-2 data was collected by 12 enumerators who had degrees in social sciences or psychology and experiences in child development assessments and participated in a 4 week training. They participated in a 4 week training Training for both Day-1 and Day-2 enumerators included theoretical instruction, mock practice, and hands-on practice with non-study participants/children in the community, under supervision. For Day-2, inter-observer reliability assessments were conducted between trainers and enumerators in which each enumerator conducted at least 10 tests. Enumerators were only allowed to participate in the main study once they achieved over 90% agreement with the trainers. During the data collection phase, a supervisor evaluated 10% of all tests to ensure satisfactory ongoing reliability (kappa > 0.90). Refresher training sessions were held in October 2020 for both data collection teams, 12 months after the initial training. Enumerators were not provided any explicit information about the nature or contents of the intervention or who received the intervention, but may have observed intervention infrastructure. Up to three attempts were made to find each household. Children who were vision or hearing impaired or had a severe developmental disability were excluded from the assessment.

### Outcomes

#### Primary outcomes.

To measure individual-level child cognitive development, we selected 9 subtests of the Wechsler Pre and Primary Scale of Intelligence – Fourth Edition (WPPSI-IV) developed in the United States [22]. This test aims to assess the intellectual ability of children aged 4 years 0 months to 7 years 7 months of age. Previous versions of WPPSI have been widely implemented in Bangladesh, and had previously been through rigorous cultural adaptation before use [23]. Our cultural adaptations focused on modifying individual items and pictures that were unfamiliar in the rural Bangladeshi context (e.g., we replaced the image of “Red Fire Hydrant” with “local Red Gas Cylinder”, “Life Jacket” with “Normal Jacket”, “Hour Glass” with “Table-Clock”) while maintaining the underlying intent of the question. We followed standard instructions for administration and scoring of subtests. Using the 9 subtests we constructed subscales for the Full Scale IQ (FSIQ); 3 Primary Index Scores: Verbal Comprehension Index (VCI), Fluid Reasoning Index (FRI), Working Memory Index (WMI); 3 Ancillary Index Score: General Ability Index (GAI), Nonverbal Index (NVI), and the Cognitive Proficiency Index (CPI) (details in Box A in S1 Text).

To assess children’s fine motor ability we used three measures of manual dexterity following the second edition of the Movement Assessment Battery for Children [24]. These tests have previously been used in Bangladesh [25]. To assess children’s behavior we administered the parent-reported Strengths and Difficulties Questionnaire (SDQ) [26]. The SDQ includes 25 questions about child attributes across five subscales: emotional symptoms, conduct problems, hyperactivity, peer problems, and prosocial behavior. The SDQ results in two measures of behavior, the total difficulties score, which is calculated based on four subscales (omitting the prosocial behavior subscale), and the stand-alone prosocial behavior score. The standard Bengali language (Bangla) version of SDQ has been applied previously in Bangladesh [27]. We assessed three areas of children’s executive functioning by selecting tests from neuropsychological and preschool assessment tools after piloting on 50 children [28,29]. We piloted a battery of executive function tests previously implemented in South Asia and selected the tests that showed variability for children of the age group assessed in the current study. Working memory, attention, and memory (short and delayed) were evaluated by forward word span (incorrectly labeled as digit span in the pre-analysis plan), Corsi blocks, and a narrative memory test, respectively. Finally, we evaluated academic achievement (reading, spelling, and mathematics) with a locally developed tool based on the Wide Range Achievement Test [30], which consists of reading, spelling, and arithmetic questions in Bengali, ranked with increasing difficulty. This tool has previously been applied in Bangladesh with primary school children [31]. We internally age-standardized all primary outcomes to the control group separately for data collected prior to the COVID pandemic and data collected during the COVID pandemic. We used local mean standardization with 2-month age bands. We had originally planned to adopt 4-month age bands but had sufficient sample size that allowed reduction in the size of the age band. To construct WPPSI-IV and fine motor subtest scores, we first internally standardized each subscale, then averaged the internally standardized scores across relevant susbscales for each outcome, and finally constructed a *z*-score with respect to the control group. We conducted supplementary analyses with externally standardized scores for the WPPSI-IV subscales.

#### Secondary outcomes.

We measured maternal depressive symptoms of mothers with the 20-item Center for Epidemiologic Studies Depression Scale (CES-D) [32]. The 20-item CES-D has been applied widely in Bangladesh and other South-East Asian countries [33]. We evaluated the home environment and amount of stimulation at home with an adapted version of the Middle childhood Home Observation Measurement (HOME), which has been implemented previously in Bangladesh [31,34].

#### Other measures.

We collected information on the sustained presence of technologies (e.g., handwashing stations, latrines) distributed during the initial intervention following protocols used at the 1 and 2 year follow-up time points [20].

### Statistical analysis

The sample size for the original trial was calculated to detect a difference of 0.15 in length-for-age *Z* score (LAZ) when comparing each intervention arm to the control, accounting for repeated measures within clusters [21]. Assuming a within-cluster correlation coefficient of 0.07 (the correlation coefficient for the cognitive outcomes from the 2-year assessment), attrition of 3 of the originally enrolled 8 children per cluster (for a total of 3,600 children), 90 clusters per intervention arm, and 180 clusters in the control arm, we estimated that have 80% power to detect an effect size of 0.18 standard deviations between each intervention arm and the double-sized control arm.

In this follow-up, we conducted separate analyses for each outcome, comparing the control arm to each intervention arm. In addition, we compared the WSH + N versus Nutrition, and WSH + N versus WSH to isolate the additive effects of WSH and N, respectively. We used generalized linear models for all analyses because we aimed to estimate marginal effect estimates, and wanted estimates to be comparable with the child development analysis previously conducted at endline [13]. We first calculated unadjusted mean differences using generalized linear models with an identity link function. We then calculated adjusted mean differences controlling for time of assessment (pre-COVID or during COVID) and concurrent child age along with a set of prognostic baseline characteristics. To account for the block-level cluster randomization in the original study design, all models included an indicator variable for block membership, and Huber-White robust standard errors were used to adjust for clustering at the block-level [20]. *P*-values were not adjusted for multiple comparisons; instead outcomes were pre-specified, and the focus was placed on the magnitude of the effect estimates [35]. The covariates for potential inclusion in each model were assessed with a likelihood ratio test, and covariates with *p* > 0.20 were included. All analyses were intention-to-treat and conducted within the sample assessed at the 7-year follow-up.

We conducted subgroup analyses by maternal primary education status at baseline, child sex at birth, measurement before or during the COVID-19 pandemic, and socioeconomic status at baseline (wealth index split at the median). We had originally planned to conduct additional stratified analyses by household distance from Dhaka (split at the median distance) but found that distance from Dhaka was strongly correlated with measurement time period (pre-COVID versus during COVID), so we did not conduct this analysis. We had also originally planned to do an additional subgroup analysis by child age at assessment, but the lack of full age overlap across the pre-COVID versus during COVID assessment periods made this not viable as an independent analysis. Subgroup analyses were conducted by including an interaction term between the treatment indicator and the subgroup of interest.

All analyses were done in R, version 4.4.3 [36]. The pre-analysis plan for this study is posted on OSF (https://osf.io/rwmg4) and included in Supporting Information (S1 File). Data from the follow-up were only linked to baseline intervention arm data following the completion of the data collection and posting of the pre-analysis plan.

The original trial (NCT01590095) and follow-up data collection (NCT04443855) are both registered with ClinicalTrials.gov.

## Results

**Fig 1:**
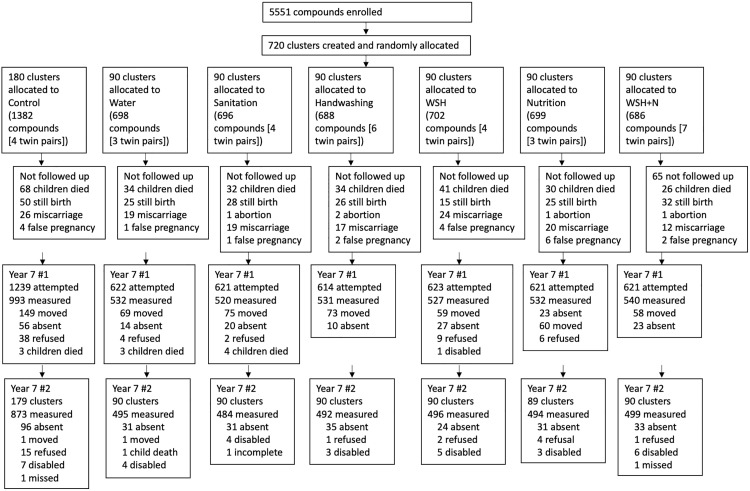
Flow diagram. Follow-up #1: Included water, sanitation, and handwashing infrastructure, the stimulating home environment, child behavior, and anthropometry; Follow-up #2: included all direct cognitive, motor and school achievement assessments, and maternal mental health.

Between September 2019 and February 2021, enumerators attempted to locate 4,961 children in 4,932 households (Fig 1). Complete assessments for both days were performed for 3,833 children in 3,812 households (21 pairs of twins). The children who had both assessments represented 69% of the initially enrolled sample, or 77% of the sample that was attempted at follow-up which included all live births not known to have passed away. Participants followed up were similar to those who were lost to follow-up across a range of characteristics (Table B in S1 Text).

A total of 4,175 children (84% of those attempted) completed the first assessment, with the most common reasons for not completing the assessment that the family had migrated (*n* = 543, 11% of those attempted) or the child was absent from household (*n* = 173, 3%) (Fig 1). At the second visit, which included the child development assessment, an additional 342 (8% of the sample attempted) did not complete the assessment, with the most common reason that the child was absent from the household at the second visit (*n* = 281, 7% of those attempted for visit 2). Common reasons for absence from the household during the second visit were holidays, disruptions due to the COVID shut down, and the post-COVID reopening of residential schools and madrasas. Assessments were paused during the initial phases of the COVID-19 pandemic from March 15th to October 13th, 2020. Due to COVID-19 pandemic related disruptions, as well as households that were unavailable for a second assessment within 3 weeks, 23% of assessments (*n* = 897) were conducted with greater than 3 weeks between days 1 and 2.

Baseline characteristics of the sample are similar across study arms for participants who received both assessments at the 7-year follow-up (Table 1). Children with child development assessments had a mean age of 83.4 months (Range: 74.4–92.0, SD: 3.5) at the child development assessment.

**Table 1:** Baseline characteristics of the re-enrolled populations.

	Control	Water	Sanitation	Handwashing	WSH	Nutrition	WSH + N
**No. of clusters**	179	90	90	90	90	89	90
	**(*N* = 873)**	**(*N* = 495)**	**(*N* = 484)**	**(*N* = 492)**	**(*N* = 496)**	**(*N* = 494)**	**(*N* = 499)**
**Maternal**							
Age (years)	24.0 (5.0)	24.0 (5.3)	24.2 (5.0)	23.9 (5.4)	24.7 (5.7)	23.9 (5.2)	24.1 (5.5)
Years of education	5.7 (3.4)	5.7 (3.4)	6.0 (3.5)	5.9 (3.3)	5.9 (3.3)	5.8 (3.5)	5.6 (3.5)
**Paternal**							
Years of education	4.8 (3.9)	4.8 (4.0)	5.2 (4.1)	4.7 (4.1)	5.0 (4.2)	4.7 (3.9)	4.7 (3.9)
Works in agriculture	282 (32%)	166 (34%)	150 (31%)	190 (39%)	161 (32%)	164 (33%)	148 (30%)
**Household**							
Number of persons	4.7 (2.1)	4.7 (2.3)	4.8 (2.1)	4.8 (2.2)	4.6 (2.0)	4.6 (2.0)	4.8 (2.1)
Has electricity	518 (59%)	305 (62%)	297 (61%)	292 (59%)	319 (64%)	299 (61%)	301 (60%)
Has a cement floor	96 (11%)	56 (11%)	61 (13%)	37 (8%)	56 (11%)	45 (9%)	61 (12%)
Acres of agricultural land owned	0.1 (0.2)	0.1 (0.2)	0.2 (0.3)	0.2 (0.2)	0.2 (0.2)	0.2 (0.3)	0.1 (0.4)
**Drinking Water**							
Tubewell primary water source	669 (77%)	360 (73%)	362 (75%)	341 (69%)	389 (78%)	370 (75%)	363 (73%)
Stored water observed at home	421 (48%)	249 (50%)	230 (48%)	242 (49%)	217 (44%)	212 (43%)	236 (47%)
**Sanitation**							
Daily defecation in the open							
Adult men	63 (7%)	24 (5%)	33 (7%)	46 (9%)	31 (6%)	37 (8%)	38 (8%)
Adult women	46 (5%)	13 (3%)	22 (5%)	21 (4%)	18 (4%)	23 (5%)	18 (4%)
**Latrine**							
Owned	452 (52%)	263 (53%)	262 (54%)	271 (55%)	263 (53%)	266 (54%)	268 (54%)
Concrete slab	784 (90%)	461 (93%)	427 (88%)	443 (90%)	451 (91%)	445 (90%)	453 (91%)
Functional water seal	226 (26%)	136 (27%)	126 (26%)	124 (25%)	104 (21%)	135 (27%)	112 (23%)
Visible stool on slab or floor	405 (46%)	252 (51%)	236 (49%)	239 (49%)	209 (42%)	229 (46%)	219 (44%)
Owned a potty	32 (4%)	17 (3%)	19 (4%)	23 (5%)	20 (4%)	28 (6%)	18 (4%)
Human feces observed in the							
House	79 (9%)	47 (10%)	43 (9%)	54 (11%)	32 (6%)	40 (8%)	33 (7%)
Child’s play area	12 (1%)	6 (1%)	6 (1%)	7 (1%)	3 (1%)	5 (1%)	5 (1%)
**Handwashing**							
Within 6 steps of latrine							
Has water	114 (13%)	63 (13%)	59 (12%)	42 (9%)	42 (8%)	44 (9%)	60 (12%)
Has soap	58 (7%)	34 (7%)	34 (7%)	23 (5%)	25 (5%)	24 (5%)	27 (5%)
Within 6 steps of kitchen							
Has water	75 (9%)	32 (6%)	35 (7%)	25 (5%)	40 (8%)	45 (9%)	45 (9%)
Has soap	21 (2%)	10 (2%)	11 (2%)	6 (1%)	11 (2%)	17 (3%)	15 (3%)
**Nutrition**							
Food secure	595 (68%)	352 (71%)	335 (69%)	342 (70%)	337 (68%)	337 (68%)	355 (71%)

Data are *n* (%) or mean (SD); N per arm includes 21 sets of twins. This table presents data from participants re-enrolled with data collected on both data collection days. For baseline characteristics of all children enrolled in the first day (some lost on the second day), see Table C in S1 Text. Missing data for the following variables: human feces observed in the house (5 missing), and child’s play area (7 missing), Owned a potty (2 missing), Daily defection in the open for adult men (73 missing) and adult women (3 missing), Acres of agriculture land owned (178 missing), Paternal works in agriculture (1 missing), Maternal age (13 missing).

At the 7-year follow-up, households in intervention arms that received the sanitation intervention were more likely to have a latrine with a functional water seal (Sanitation: 83% (*n* = 393), WSH: 78% (*n* = 383), and WSH + N: 83% (*n* = 410)) compared to the control arm (48%, *n* = 421)) and other arms without sanitation (Water: 51% (*n* = 250), Handwashing: 50% (*n* = 246), Nutrition: 51% (*n* = 249)) (Table 2). The proportion of households in the Sanitation arms with a latrine with a functional water seal was lower than at the 2-year endline. Other indicators of intervention adherence including the presence of stored drinking water, visible feces on the latrine slab or floor, and the presence of soap at the handwashing station were not substantially different across arms, including the control arm. Intra-cluster correlations ranged from <0.01 to 0.18 for primary outcomes, and <0.01 to 0.31 for secondary outcomes (Table D in S1 Text).

**Table 2:** Sustained adoption of behavioral recommendations and continued presence of technologies promoted or distributed during the period of project implementation.

	Control	Water	Sanitation	Handwashing	WSH	Nutrition	WSH + N
**Number of compounds measured (*N*)**							
Enrollment	1,382 (100%)	698 (100%)	696 (100%)	688 (100%)	702 (100%)	699 (100%)	686 (100%)
Year 1	1,151 (83%)	611 (88%)	583 (84%)	585 (85%)	605 (86%)	581 (83%)	600 (87%)
Year 2	1,138 (82%)	598 (86%)	585 (84%)	570 (83%)	588 (84%)	574 (82%)	586 (85%)
Year 7	872 (63%)	493 (71%)	479 (69%)	489 (71%)	492 (70%)	491 (70%)	495 (72%)
**Stored drinking water (%)**							
Enrollment	666 (48%)	353 (51%)	341 (49%)	347 (50%)	304 (43%)	301 (43%)	331 (48%)
Year 1	503 (44%)	587 (96%)	245 (42%)	266 (45%)	588 (97%)	229 (39%)	577 (96%)
Year 2	485 (43%)	567 (95%)	260 (44%)	267 (47%)	558 (95%)	225 (39%)	569 (97%)
Year 7	326 (37%)	197 (40%)	180 (38%)	160 (33%)	193 (39%)	192 (39%)	202 (41%)
**Latrine with a functional water seal (%)**							
Enrollment	358 (26%)	183 (26%)	177 (25%)	162 (24%)	152 (22%)	183 (26%)	155 (23%)
Year 1	308 (27%)	151 (25%)	554 (95%)	144 (25%)	573 (95%)	149 (26%)	564 (94%)
Year 2	324 (28%)	184 (31%)	568 (97%)	165 (29%)	567 (96%)	163 (28%)	561 (96%)
Year 7	421 (48%)	250 (51%)	398 (83%)	246 (50%)	383 (78%)	249 (51%)	410 (83%)
**Latrine with no visible feces on latrine slab or floor (%)**							
Enrollment	625 (48%)	350 (53%)	332 (52%)	335 (52%)	289 (44%)	331 (51%)	298 (46%)
Year 1	658 (60%)	358 (61%)	516 (89%)	324 (58%)	522 (86%)	333 (60%)	527 (88%)
Year 2	612 (56%)	338 (58%)	502 (86%)	324 (60%)	484 (82%)	313 (58%)	495 (85%)
Year 7	501 (57%)	287 (58%)	317 (66%)	270 (56%)	295 (60%)	294 (60%)	328 (66%)
**Handwashing location with soap (%)**							
Enrollment	294 (21%)	153 (22%)	155 (22%)	134 (19%)	155 (22%)	152 (22%)	149 (22%)
Year 1	283 (25%)	165 (27%)	158 (27%)	533 (91%)	546 (90%)	172 (30%)	536 (89%)
Year 2	320 (28%)	177 (30%)	180 (31%)	527 (92%)	531 (90%)	195 (34%)	540 (92%)
Year 7	352 (40%)	197 (40%)	184 (38%)	194 (40%)	236 (48%)	196 (40%)	245 (49%)

Data are *n* (%); Intervention began after enrollment and continued until the Year 2 assessment. No intervention was implemented between Year 2 and Year 7. Year 7 data presented in this table are from participants who completed both days of data collection at 7 years of age. Enrollment, Year 1, and Year 2 measures differ slightly from those that were previously reported [20] for the latrine with functional water seal and handwashing location with soap indicators (due to an updated definition for those classified as “no”, see current definitions below).

Stored drinking water: the calculation of this indicator differs between the first three assessment time points and year 7. At the earlier time points, this was determined by a demonstration of where water for children was obtained, and was scored as yes if it came from a storage container or directly from a water filter. At 7 years this was scored as yes if the response to “Is there any stored water in your household now?” was yes and observed.

Latrine with a functional water seal: participants with an observed flush latrine with a functional water seal were classified as “yes” on this indicator. Participants with no latrine, no flush latrine, a flush latrine without a functional water seal, a latrine that was not observed, or the observer could not tell if there was a functional water seal were classified as “no” on this indicator.

Latrine with no visible feces on latrine slab or floor: participants who did not have a latrine, had a latrine that was not observed, or for whom the response was “not applicable” to the observation of stool on the slab or floor were categorized as missing for this indicator (missing at enrollment: *n* = 349; Year 1: *n* = 140; Year 2: *n* = 136; Year 7: = 23)

Handwashing location with soap: participants with an observed handwashing station that had soap available were classified as “yes” for this indicator. Participants with no specific location for a handwashing station, or a handwashing station observed with no soap available classified as “no”.

### WPPSI-IV.

**Fig 2:**
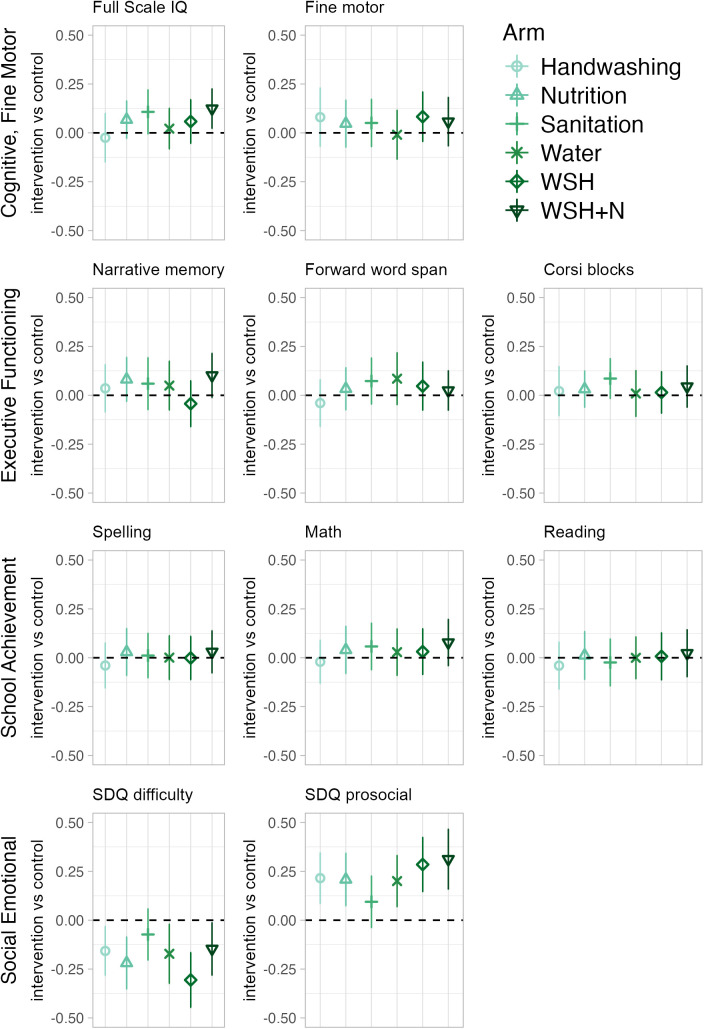
Differences in child development and school achievement in control vs. intervention arm. Point estimates reflect the mean difference between each intervention arm and the control arm from generalized linear models that adjust for child age, measurement period (pre-COVID or during COVID), and prognostic baseline control variables (significant at *p* < 0.20 in a likelihood ratio test) to increase precision (for example of included control variables see Table G in S1 Text). All outcomes are presented as internally standardized *z*-scores, and all results account for the clustered study design through Huber–White robust standard errors clustered at the block-level. Bars represent 95% confidence intervals. Full Scale IQ is a composite measure from the WPPSI-IV, SDQ: strengths and difficulties questionnaire, Narrative memory: Sum of free and cued recall scores from a narrative memory test.

Compared to children in the control arm, children in the WSH + N arm had better FSIQ scores (adjusted mean difference: 0.12 (95% CI: 0.02, 0.23)) (Fig 2, Table E in S1 Text). Children in all other arms did not have differences in FSIQ scores compared to children in the control arm that were statistically significant at *p* < 0.05 (Fig 2, Table E in S1 Text). Compared to children in the control arm, children had better FRI scores in the WSH + N (0.15 (0.03, 0.27)), Sanitation (0.12 (0.00, 0.24)), and Nutrition arms (0.12 (0.01, 0.23)) (Fig A in S1 Text, Table E in S1 Text). Children also had better WPPSI-IV GAI scores in the WSH + N (0.12 (0.03, 0.22)), Sanitation (0.12 (0.01, 0.23)), and Nutrition (0.11 (0.01, 0.20)) arms, better NVI scores in the WSH + N (0.13 (0.03, 0.24)) and Sanitation (0.11 (0.00, 0.22)) arms, and better VCI scores in the nutrition arm (0.10 (0.00, 0.20)) (Fig A in S1 Text, Table E in S1 Text). There were no differences between any intervention arm and the control arm for the CPI or WMI scores (Fig A in S1 Text, Table E in S1 Text).

### Fine motor, executive functioning, academic achievement, difficult, and prosocial behavior.

For the majority of fine motor, executive functioning, and academic achievement outcomes adjusted differences between children in intervention arms compared to control were either close to null (under 0.05) or indicated small (0.05–0.11) improvements compared to the control arms. For example, for executive functioning, there were small improvements in outcomes for children in the Nutrition, Sanitation, Water, and WSH + N arms for one or two of the executive functioning tests (largest effect: 0.10 (−0.01, 0.21 for WSH + N versus control on the narrative memory task) (Fig 2, Table H in S1 Text, Fig B in S1 Text). The measure of socioemotional development showed that difficult behaviors were reduced in all intervention arms except for Sanitation (largest effect: −0.31 (−0.45, −0.17) for WSH versus control), and prosocial behavior scores were higher for children in all intervention arms except for the Sanitation arm compared to the control arm (Water: 0.20 (0.07, 0.33), Handwashing: 0.22 (0.09, 0.34), WSH: 0.28 (0.15, 0.42), Nutrition: 0.21 (0.07, 0.34), WSH + N: 0.31 (0.16, 0.46) (Fig 2, Table H in S1 Text). When we broke down the total difficulties score into its four subscales, the Peer relationship problems subscale had the smallest magnitude of effects across arms (between 0.00 (−0.17, 0.16) for Handwashing to −0.08 (−0.24, 0.08) for WSH + N), and the Conduct problems, Hyperactivity/inattention, and Emotional symptoms subscales had similar magnitudes of effects across arms (between −0.04 (−0.16, 0.07) for Sanitation in Conduct to −0.24 (−0.38, −0.10) for WSH in Conduct and −0.24 (−0.38, −0.11) for Nutrition in Emotion) (Table J in S1 text)

### Secondary outcomes.

**Fig 3:**
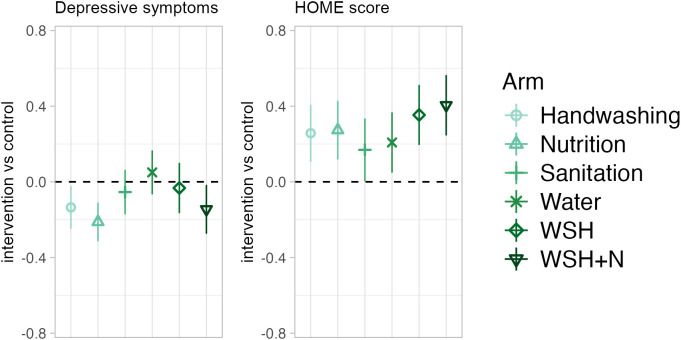
Differences in secondary outcomes by arm. Point estimates reflect the mean difference between each intervention arm and the control arm from generalized linear models that adjust for child age, measurement period (pre-COVID or during COVID), and prognostic baseline control variables (significant at *p* < 0.20 in a likelihood ratio test) to increase precision (for example, of included control variables see Table G in S1 Text). All outcomes are presented as internally standardized *z*-scores, and all results account for the clustered study design through Huber–White robust standard errors clustered at the block-level. Bars represent 95% confidence intervals. Depressive symptoms are maternal depressive symptoms measured by the Center for Epidemiologic Studies 20-question depression measure (CES-D); HOME: Middle childhood Home Observation Measurement of the Environment.

Caregivers had fewer depressive symptoms in the WSH + N (−0.15 (−0.27, −0.02)), Handwashing (−0.14 (−0.24, −0.03)), and Nutrition (−0.21 (−0.31, −0.11)) arms compared with caregivers in the control group. Children in all intervention arms had more stimulating home environments (Water: 0.21 (0.05, 0.36), Sanitation: 0.17 (0.01, 0.33), Handwashing: 0.26 (0.11, 0.40), WSH: 0.36 (0.20, 0.51), Nutrition: 0.27 (0.12, 0.42), WSH + N: 0.40 (0.25, 0.56)) than children in the control group (Fig 3, Table K in S1 Text).

### Subgroup analyses.

**Fig 4:**
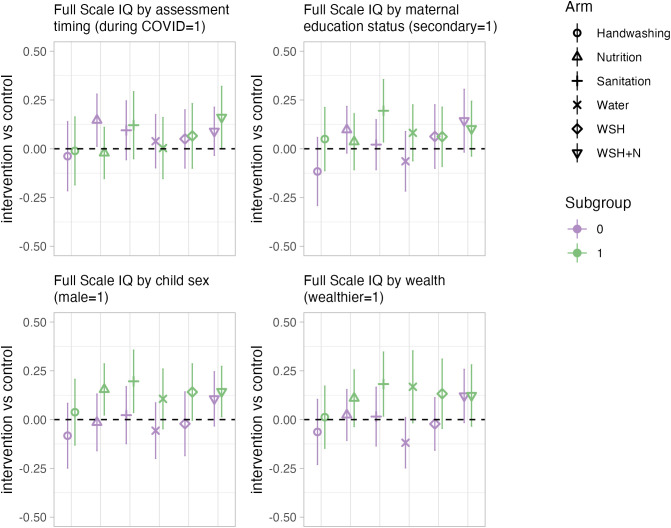
Subgroup analysis for FSIQ outcome. Point estimates reflect the mean difference between each intervention arm and the control arm in each subgroup, from generalized linear models that adjust for child age, measurement period (pre-COVID or during COVID), and prognostic baseline control variables (significant at *p* < 0.20 in a likelihood ratio test) to increase precision (for example, of included control variables see Table G in S1 Text). All outcomes are presented as internally standardized *z*-scores, and all results account for the clustered study design through Huber–White robust standard errors clustered at the block-level. Bars represent 95% confidence intervals. Full Scale IQ is a composite measure from the WPPSI-IV.

In subgroup analyses, there was no consistent heterogeneity in intervention effects on FSIQ by assessment timing (prior to or during the COVID pandemic), or maternal education status (mother had completed secondary school prior to pregnancy) (Fig 4). However, families with higher wealth indices at baseline, and those with male children tended to have larger effect sizes on the FSIQ (Fig 4). For SDQ prosocial and total difficulties measures, there were larger impacts for children assessed during the COVID-19 pandemic (Figs C and D in S1 Text). For school achievement, despite the lack of average impact, there were larger intervention impacts on reading, math, and spelling for wealthier households and male children (Figs F, G, H in S1 Text). There were no consistent patterns across other outcomes in heterogeneity analyses (Figs E, I–M in S1 Text). There were also no consistent differences between WSH + N and nutrition or WSH + N and WSH across outcomes (Tables E, H, L in S1 Text).

## Discussion

This medium-term follow-up study showed that 5 years after intervention completion, single or combined water, handwashing, sanitation, and nutrition interventions had small but significant sustained impacts on one or more domains of child development, the home environment, and caregiver mental well-being. Seven-year-old children who had received the WSN + N, Nutrition, or Sanitation intervention in-utero and as babies had improved cognitive development in three of the six WPPSI-IV subscales when compared to children in the control arm. Children in all arms except for the Sanitation arm had reduced difficult behaviors and improved prosocial behaviors, and children in all intervention arms had more stimulating home environments compared to the control arm. Caregiver-related outcomes were also improved, with caregivers in the WSH + N, Handwashing, and Nutrition arms having fewer depressive symptoms. For the majority of intervention arms, there were small and likely not clinically meaningful improvements in fine motor, executive functioning, and academic achievement. We found that for a few outcomes results tended to differ by subgroup status. For FSIQ, impacts tended to be larger in families with higher baseline wealth and among male children. For socioemotional outcomes, children assessed during the COVID-19 pandemic had larger impacts. For reading, math, and spelling achievement, there were small improvements in wealthier households and male children in some intervention arms, but not in less wealthy and female children. Impacts across other outcomes did not substantially differ by assessment timing (prior to or during the COVID-19 pandemic), maternal education status, child sex, or wealth status.

The interventions in the WASH-Benefits Bangladesh study were designed to promote the adoption of the recommended technologies and behaviors during the first 2 years of life, throughout the 2-year intervention period. During this time households received support from health workers to implement, and resolve barriers to, the recommended behaviors and technologies. At the 7-year follow-up, the presence of a latrine with a functional water seal remained markedly higher in the arms that received the sanitation intervention compared to those who did not (83% for WSH + N and 50% for the control arm). For measures of adherence for the water (stored drinking water) and handwashing (presence of a handwashing location with soap) arms, however, sustained impacts on adherence measures were not present. The sustained presence of a handwashing location with soap (e.g., 40% in the handwashing arm) differs substantially from results from a handwashing intervention in Karachi, Pakistan that found 97% of intervention households still had handwashing stations 5 years following intervention completion [37]. These two interventions differed substantially in their contexts: In the urban squatter settlements where the Karachi intervention took place, the handwashing stations were easier to maintain than in the rural villages where the WASH-Benefits intervention took place. Most households in the Karachi study had overhead water tanks that were accessed through a faucet, and it was straightforward to put a soap dish with soap next to the tap; in the rural WASH-Benefits Bangladesh villages soapy water was an innovation and was not as convenient as water obtained from a tubewell that needed to be pumped. Interventions that aim to promote long-term changes in behavior would require adaptations; one such adaptation could be incorporating structural changes to facilitate ease and convenience of the promoted behaviors as well as reductions in the required maintenance of infrastructure for sustained use.

Our analysis was unique in that it followed up children who had received early WASH interventions that improved child development at 2 years of age [13]. Two other randomized controlled trials have evaluated the impact of early WASH interventions on early child development outcomes immediately post-intervention with one following up children at 7 years of age, neither found any improvements for children who received the interventions [14,15,17]. The previously discussed study in Pakistan evaluated later, but not immediate impacts of a handwashing and water intervention on child development [18]. In Pakistan, children who were randomized to receive either hand washing promotion or hand washing promotion plus water treatment interventions during the first 30 months of life subsequently were followed up between 5 and 7 years of age. Children who received the intervention had improvements on a composite measure of development equivalent to an effect size of 0.4 standard deviations. Other interventions have evaluated a combination of sanitation or handwashing interventions with stimulation on outcomes later in childhood, and found improved child development and caregiver mental health among families who received the intervention [38,39]. However, these interventions were unable to untangle the impacts of the water, sanitation, and handwashing component from the psychosocial stimulation component.

In contrast to the evidence for early WASH interventions, there is consistent existing evidence for the impact of early nutrition supplementation on short-term early child development. A recent systematic review and meta-analyses [8], examined the impact of early small-quantity lipid-based multiple micronutrient supplements delivered within the first 1,000 days on short-term child development outcomes in LMICs and found that 11 out of 13 interventions showed moderate, post-intervention benefits to language, socioemotional, motor, and executive function. However, few of these studies followed up with the children in the study to assess outcomes later in childhood. In rural Pakistan, a nutrition education and multiple micronutrient powder supplementation intervention for children 6–24 months of age improved motor development, but not WPPSI-III FSIQ, executive functioning, pre-academic skills or behavioral problems at 4 years of age [40]. In Ghana, lipid-based nutrient supplements provided between 6–24 months reduced socioemotional difficulties among children at 4–6 years of age, but didn’t impact cognitive or fine motor development outcomes [41]. These impacts on socioemotional difficulties did not persist to 9–11 years of age [42]. A micronutrient supplementation trial in Indonesia improved procedural memory in children at 9–12 years of age, and children born to anemic mothers had improved general intellectual ability [43]. Finally, a small factorial designed nutrition and stimulation intervention delivered in Jamaica found no impact of 2 years of early supplementation with milk-based formula on child development outcomes at 7–8 years of age, except in a subset of children of mothers who had higher verbal intelligence quotients; no impact was found from supplementation on child development outcomes at later time points [44].

In this follow-up study, we found small but sustained benefits in Full Scale IQ for children in the Sanitation and WSH + N arms. We also found improvements in three out of six total WPPSI sub-domains for the WSH + N, Nutrition, and Sanitation arms, fewer difficult behaviors, and improved prosocial behaviors in all intervention arms except for Sanitation. The intervention was provided during the first 1,000 days of life, a critical and sensitive window of development [5,45]. There are two primary pathways through which we hypothesize these interventions could have had sustained impacts. The first is through reduced enteric infections, as the initial interventions reduced diarrhea at 2 years of age in all intervention arms except for the Water arm [20]. Inflammation associated with diarrhea limits the absorption of key nutrients, and repeated episodes of diarrhea before age two are associated with poorer cognitive development outcomes, so this may also be a mechanism by which the early intervention had sustained effects [46,47]. Additionally, the two previous WASH interventions that did not find impacts on child development at intervention completion also did not find impacts on diarrhea [48,49]. Another potential pathway of intervention effect could be through support of caregivers and improved caregiving practices, which were higher in the WASH-Benefits Bangladesh intervention than in the previous WASH interventions. In all active intervention arms, community health promotors were instructed to visited intervention households weekly for the first 6 months, and then every 2 weeks for the subsequent 18 months, the actual number of visits per month was an average of 5–7 throughout the intervention period [50]. These visits started during the 1st and 2nd trimester of pregnancy and continued for almost 2 years after birth. Although support for caregiver mental well-being or child stimulation was not explicitly provided, maternal depressive symptoms were significantly reduced in the WSH + N, H, and N arms, and the stimulating home environment was improved in all intervention arms. Caregiver mental health and the caregiving environment were also improved at the 2-year endline assessment reported previously [13]. The impacts on child development may be due to increased attention to the child and social support provided to the caregiver during the frequent household visits leading to both improved mental health of caregivers and more attention to and investment in the child’s physical and social environment. Improvements in home stimulation is a target of many child development-specific interventions [51].

While we found impacts on some measures of child development, for multiple measures we identified small or no impacts. For example, we found impacts on narrative memory for children in the WSH + N arm, but did not find any impacts for any other intervention arms or any other measures of executive functioning. To evaluate executive functioning, we chose tests that focused on memory and attention but did not include a test that evaluated inhibitory control as it was not adequately adapted to the population (we observed ceiling effects during piloting). Tablet-based assessment of inhibitory control has promise for future assessments in this age group [52], but was not feasible given the logistical constraints of working in rural Bangladesh. The chosen tests may not have been sensitive enough to evaluate the intervention’s impact. We also did not find impacts on fine motor or academic achievement. A lack of effects on school achievement may be because most children have only been exposed to one or fewer years of formal schooling at this age. Given that intervention effects did not differ between children assessed prior to and during the COVID-19 pandemic, educational interruption during the early COVID-19 pandemic period was not likely to have affected this finding.

Our study has several strengths including its basis on a large RCT design with well-balanced intervention arms and a double-sized control. We evaluated both individual and combined interventions to understand the unique and combined impacts of each intervention. The enumerators went through a rigorous training for developmental assessments and monitored for 10% quality-check throughout the study period. There were direct assessments for children’s development and observation for the home environment, and all analyses were pre-specified. Our outcome measures had good psychometric properties.

A key weakness of the study is that we were unable to assess 31% of initially enrolled participants on the full set of assessments (23% of live births from the original study that had not been known to have passed away since the study started). However, we did not find major differences in baseline characteristics between participants who lost to follow and those followed up, reducing the potential bias induced by loss to follow-up. In addition, the applied child development assessment tools were not initially developed or standardized for use in LMIC settings. We did, however, culturally adapt all measures for use in our study setting in Bangladesh and conducted rigorous piloting. We also used internally standardized *z*-scores instead of standardized scores from external high-income populations. Further, our data collection period was interrupted by the COVID-19 pandemic, and paused between March and September 2020, which may have affected the results (see S2 Checklist). We adjusted for whether the assessment occurred before or during the COVID-19 pandemic within the standardization of outcomes, controlled for it in all analyses, and conducted subgroup analyses to examine differences. Finally, we examined multiple outcomes and acknowledge that no adjustment was made for multiple comparisons. Instead of focusing on *p*-values we focused on interpreting the magnitude and direction of coefficients across outcomes [53].

We found that an early water, sanitation, handwashing, and nutrition interventions had sustained impacts on child development, caregiver mental health, and the stimulating home environment. Future work to elucidate the mechanisms of these impacts will help to isolate the key components of these interventions.

## Supporting information

S1 Text **Box A**. Details on WPPSI-IV tests and indices. **Table A**. WASH-B intervention components by intervention arm. **Table B**. Comparing baseline characteristics for assessed vs. lost participants at the 5-year follow-up. **Table C**. Baseline characteristics of the re-enrolled populations (Day 1 sample). **Table D**. Intra-cluster correlation by outcome. **Table E**. WPPSI results. **Table F**. WPPSI results: Adjusted analysis sample size. **Table G**. Control variables included in adjusted regression models. **Table H**. Fine motor, Executive functioning, School achievement, SDQ results. **Table I**. Fine motor, Executive functioning, School achievement, SDQ results: adjusted analysis sample size. **Table J**. SDQ difficulties subscale results. **Table K**. Secondary outcome results. **Table L**. Secondary outcome results: adjusted analysis n’s. **Fig A**. WPPSI index scores. **Fig B**. Expanded narrative memory results. **Fig C**. Subgroup analysis for SDQ prosocial outcome. **Fig D**. Subgroup analysis for SDQ difficulties. **Fig E**. Subgroup analysis for Fine motor outcome. **Fig F**. Subgroup analysis for Math achievement. **Fig G**. Subgroup analysis for Reading achievement. **Fig H**. Subgroup analysis for Spelling achievement. **Fig I**. Subgroup for Narrative memory. **Fig J**. Subgroup for Corsi blocks. **Fig K**. Subgroup for Forward word span. **Fig L**. Subgroup analysis for HOME. **Fig M**. Subgroup analysis for Maternal Depressive symptoms.
(DOCX)

S1 Checklist CONSORT checklist. Completed CONSORT 2010 extension for cluster-randomized trials checklist (https://doi.org/10.1136/bmj.e5661). This checklist is licensed under the Creative Commons Attribution 4.0 International License.
(DOCX)

S2 Checklist CONSERVE checklist. Completed CONSERVE-CONSORT checklist. This file reports the changes made to the study as a result of the COVID-19 pandemic as per the CONSERVE-CONSORT checklist.
(DOCX)

S1 File Pre-analysis plan. (PDF)

## Abbreviations

CES-D: Center for Epidemiologic Studies Depression Scale
CPI: Cognitive Proficiency Index
cRCT: cluster-randomized controlled trial
FRI: Fluid Reasoning Index
FSIQ: Full Scale IQ
GAI: General Ability Index
HOME: Middle childhood Home Observation Measurement of the Environment
LAZ: length-for-age *Z* score
LMICs: low- and middle-income countries
NVI: Nonverbal Index
SDQ: Strengths and Difficulties Questionnaire
VCI: Verbal Comprehension Index
WASH: water, sanitation, and hygiene
WMI: Working Memory Index
WPPSI-IV: Wechsler Pre and Primary Scale of Intelligence – Fourth Edition
WSH: Combined Water, Sanitation and Hygiene intervention arm
WSH + N: Combined Water, Sanitation, Hygiene and Nutrition intervention arm
