# Supplementary material for: Effects of early water, sanitation, handwashing, and nutrition interventions on child development at school age: a follow-on study of a cluster-randomized trial in rural Bangladesh
Source: PLoS Med. 2025 Dec 16;22(12):e1004793. doi: 10.1371/journal.pmed.1004793 (PMC12707674; doi:10.1371/journal.pmed.1004793)
Supplement: S1 Text — Box A. Details on WPPSI-IV tests and indices. Table A. WASH-B intervention components by intervention arm. Table B. Comparing baseline characteristics for assessed vs. lost participants at the 5-year follow-up. Table C. Baseline characteristics of the re-enrolled populations (Day 1 sample). Table D. Intra-cluster correlation by outcome. Table E. WPPSI results. Table F. WPPSI results: Adjusted analysis sample size. Table G. Control variables included in adjusted regression models. Table H. Fine motor, Executive functioning, School achievement, SDQ results. Table I. Fine motor, Executive functioning, School achievement, SDQ results: adjusted analysis sample size. Table J. SDQ difficulties subscale results. Table K. Secondary outcome results. Table L. Secondary outcome results: adjusted analysis n’s. Fig A. WPPSI index scores. Fig B. Expanded narrative memory results. Fig C. Subgroup analysis for SDQ prosocial outcome. Fig D. Subgroup analysis for SDQ difficulties. Fig E. Subgroup analysis for Fine motor outcome. Fig F. Subgroup analysis for Math achievement. Fig G. Subgroup analysis for Reading achievement. Fig H. Subgroup analysis for Spelling achievement. Fig I. Subgroup for Narrative memory. Fig J. Subgroup for Corsi blocks. Fig K. Subgroup for Forward word span. Fig L. Subgroup analysis for HOME. Fig M. Subgroup analysis for Maternal Depressive symptoms. (DOCX) [file pmed.1004793.s001.docx]

**Supplementary Tables and Figures**

**Box A. Details on WPPSI-IV tests and indices**

| We selected 9 subtests of the Wechsler Pre and Primary School Intelligence – Fourth Edition (WPPSI-IV). Out of these, 6 were core subtests (Information, Similarities, Block Design, Matrix Reasoning, Picture Memory and Bug Search) used to construct the Full-Scale IQ (FSIQ). An additional 3 of 7 supplemental subtests (Picture Concept, Zoo Location, and Animal Coding), were selected based on the performance of the subtest in initial piloting, and also to enable the construction of three additional Primary Index Scores (Verbal Comprehension Index (VCI), Fluid Reasoning Index (FRI), Working Memory Index (WMI), and three Ancillary Index Scores (General Ability Index (GAI), Nonverbal Index (NVI), and the Cognitive Proficiency Index (CPI)). The specific tests used in each index is as follows:  Primary indices   - VCI: Information, Similarities - FRI: Matrix Reasoning, Picture Concepts - WMI: Picture Memory, Zoo Location   Ancillary Index scores   - GAI: Information, Similarities, Block Design, Matrix Reasoning - NVI: Block Design, Matrix Reasoning, Picture Concept, Picture Memory, Bug Search - CPI: Picture Memory, Zoo Location, Bug Search, Animal Coding |
| --- |

**Table A.** **WASH-B intervention components by intervention arm**

|  | **Water** | **Sanitation** | **Handwashing** | **Nutrition** | **Water, Sanitation, Handwashing** | **Water, Sanitation, Handwashing, and nutrition** |
| --- | --- | --- | --- | --- | --- | --- |
| **Technology and supplies provided** | Insulated storage container for drinking water; Aquatabs (Medentech, Ireland) | Sani-scoop; potty; double-pit pour flush improved latrine | Handwashing station; storage bottle for soapy water; laundry detergent sachets for preparation of soapy water | LNS (Nutriset, France); storage container for LNS | Same as individual water, sanitation, and handwashing interventions | Same as individual water, sanitation, handwashing, and nutrition interventions |
| **Key behavioral recommendations delivered by promoters** | Targeted children drink treated, safely stored water | Family use double pit latrines; potty train children; safely dispose of faeces into latrine or pit | Family wash hands with soap after defecation and during food preparation | Exclusive breastfeeding up to 180 days; introduce diverse complementary food at 6 months; feed LNS from 6–24 months | Same as individual water, sanitation, and handwashing interventions | Same as individual water, sanitation, handwashing, and nutrition interventions |
| **Population targeted** | Children younger than 5 years living in index households | Whole compound for latrines; index households for potty training and safe faeces disposal | Residents of index households | Index children (targeted through mother) | Same as individual water, sanitation, and handwashing interventions | Same as individual water, sanitation, handwashing, and nutrition interventions |
| **Emphasis during visits after refresher training** | Safe storage of water, children drink only treated and safely stored water | Latrine cleanliness; maintenance; pit switching | Handwashing before food preparation | Dietary diversity during complementary feeding; provide LNS even if child is unwell | Same as individual water, sanitation, and handwashing interventions | Same as individual water, sanitation, handwashing, and nutrition interventions |

LNS=lipid-based nutrient supplement. †Promoter visits were intended to teach participants how to use technologies and how to use and restock products; arrange for social support; communicate benefits of use and practice and changes in social norms; congratulate and encourage; problem-solve as needed; and inspire. Techniques used included counselling via flipcharts and cue cards, onsite demonstrations of technologies and products, video dramas, storytelling, games, and songs. Promoter’s guides detailed the visit objective, target audience, and the specific steps and materials to be used.

Table reproduced with minor adaptations from Luby et al 2018 [1], with permission.

**Table B. Comparing baseline characteristics for assessed vs. lost participants at the 5-year follow-up**

|  | **Assessed** | **LTFU^1^** |
| --- | --- | --- |
| **No. of compounds** | **(N = 3833)** | **(N = 1757)** |
| **Maternal** |  |  |
| **Years of education** | 5.8 (3.4) | 5.8 (3.5) |
| **Paternal** |  |  |
| **Years of education** | 4.8 (4.0) | 4.9 (4.1) |
| **Works in agriculture** | 1261 (33%) | 494 (28%) |
| **Household** |  |  |
| **Number of persons** | 4.7 (2.1) | 4.6 (2.3) |
| **Has electricity** | 2331 (61%) | 963 (55%) |
| **Has a cement floor** | 412 (11%) | 177 (10%) |
| **Acres of agricultural land owned** | 0.15 (0.26) | 0.14 (0.21) |
| **Drinking Water** |  |  |
| **Tubewell primary water source** | 2854 (74%) | 1283 (73%) |
| **Stored water observed at home** | 1807 (47%) | 857 (49%) |
| **Sanitation** |  |  |
| **Daily defecation in the open** |  |  |
| **Adult men** | 272 (7%) | 146 (8%) |
| **Adult women** | 161 (4%) | 76 (4%) |
| **Children: 8-<15 years** | 162 (10%) | 69 (10%) |
| **Children: 3-<8 years** | 774 (38%) | 314 (38%) |
| **Children: 0-<3 years** | 665 (84%) | 309 (83%) |
| **Latrine** |  |  |
| **Owned** | 2045 (53%) | 951 (54%) |
| **Concrete slab** | 3464 (94%) | 1551 (93%) |
| **Functional water seal** | 963 (30%) | 421 (29%) |
| **Visible stool on slab or floor** | 1789 (50%) | 793 (48%) |
| **Owned a potty** | 157 (4%) | 88 (5%) |
| **Human faeces observed in the** |  |  |
| **House** | 328 (9%) | 138 (8%) |
| **Child's play area** | 44 (1%) | 20 (1%) |
| **Handwashing** |  |  |
| **Within 6 steps of latrine** |  |  |
| **Has water** | 424 (12%) | 185 (11%) |
| **Has soap** | 225 (6%) | 109 (7%) |
| **Within 6 steps of kitchen** |  |  |
| **Has water** | 297 (9%) | 149 (9%) |
| **Has soap** | 91 (3%) | 41 (3%) |

^1^ Includes 33 participants who were followed-up and consented to participate, but were disabled and so did not complete the child development assessment

**Table C. Baseline characteristics of the re-enrolled populations (Day 1 sample)**

|  | **Control** | **Water** | **Sanitation** | **Handwashing** | **WSH** | **Nutrition** | **WSH+N** |
| --- | --- | --- | --- | --- | --- | --- | --- |
| **No. of clusters** | 180 | 90 | 90 | 90 | 90 | 90 | 90 |
|  | (N = 993) | (N = 532) | (N = 520) | (N = 531) | (N = 527) | (N = 532) | (N = 540) |
| **Maternal** |  |  |  |  |  |  |  |
| Age (years) | 23.9 (5.1) | 24.0 (5.3) | 24.1 (5.1) | 23.9 (5.3) | 24.6 (5.7) | 23.8 (5.2) | 24.1 (5.6) |
| Years of education | 5.8 (3.4) | 5.8 (3.4) | 6.0 (3.4) | 5.8 (3.3) | 5.9 (3.3) | 5.8 (3.4) | 5.6 (3.5) |
| **Paternal** |  |  |  |  |  |  |  |
| Years of education | 4.8 (3.9) | 4.9 (4.0) | 5.1 (4.1) | 4.5 (4.0) | 5.0 (4.2) | 4.8 (3.9) | 4.7 (3.9) |
| Works in agriculture | 310 (31%) | 175 (33%) | 162 (31%) | 204 (38%) | 170 (32%) | 178 (33%) | 165 (31%) |
| **Household** |  |  |  |  |  |  |  |
| Number of persons | 4.7 (2.1) | 4.7 (2.2) | 4.7 (2.1) | 4.8 (2.2) | 4.6 (2.0) | 4.6 (2.1) | 4.8 (2.1) |
| Has electricity | 580 (58%) | 333 (63%) | 320 (62%) | 314 (59%) | 334 (63%) | 325 (61%) | 328 (61%) |
| Has a cement floor | 107 (11%) | 64 (12%) | 67 (13%) | 38 (7%) | 59 (11%) | 53 (10%) | 64 (12%) |
| Acres of agricultural land owned | 0.1 (0.2) | 0.1 (0.2) | 0.1 (0.2) | 0.1 (0.2) | 0.2 (0.2) | 0.2 (0.2) | 0.1 (0.4) |
| **Drinking Water** |  |  |  |  |  |  |  |
| Tubewell primary water source | 754 (76%) | 386 (73%) | 385 (74%) | 371 (70%) | 412 (78%) | 396 (74%) | 395 (73%) |
| Stored water observed at home | 483 (49%) | 266 (50%) | 252 (48%) | 261 (49%) | 233 (44%) | 225 (42%) | 253 (47%) |
| **Sanitation** |  |  |  |  |  |  |  |
| Daily defecation in the open |  |  |  |  |  |  |  |
| Adult men | 67 (7%) | 28 (5%) | 34 (7%) | 52 (10%) | 34 (7%) | 39 (7%) | 39 (7%) |
| Adult women | 49 (5%) | 15 (3%) | 23 (4%) | 25 (5%) | 19 (4%) | 25 (5%) | 18 (3%) |
| **Latrine** |  |  |  |  |  |  |  |
| Owned | 524 (53%) | 282 (53%) | 282 (54%) | 286 (54%) | 282 (54%) | 291 (55%) | 289 (54%) |
| Concrete slab | 897 (90%) | 492 (92%) | 460 (88%) | 476 (90%) | 477 (91%) | 480 (90%) | 493 (91%) |
| Functional water seal | 251 (25%) | 143 (27%) | 134 (26%) | 129 (24%) | 113 (21%) | 145 (27%) | 129 (24%) |
| Visible stool on slab or floor | 457 (46%) | 275 (52%) | 253 (49%) | 261 (49%) | 221 (42%) | 248 (47%) | 240 (44%) |
| Owned a potty | 39 (4%) | 21 (4%) | 21 (4%) | 24 (5%) | 20 (4%) | 29 (5%) | 22 (4%) |
| Human feces observed in the |  |  |  |  |  |  |  |
| House | 86 (9%) | 49 (9%) | 45 (9%) | 57 (11%) | 38 (7%) | 45 (8%) | 37 (7%) |
| Child's play area | 15 (2%) | 6 (1%) | 6 (1%) | 7 (1%) | 4 (1%) | 7 (1%) | 6 (1%) |
| **Handwashing** |  |  |  |  |  |  |  |
| Within 6 steps of latrine |  |  |  |  |  |  |  |
| Has water | 131 (13%) | 69 (13%) | 64 (12%) | 44 (8%) | 45 (9%) | 47 (9%) | 64 (12%) |
| Has soap | 64 (6%) | 38 (7%) | 37 (7%) | 23 (4%) | 28 (5%) | 24 (5%) | 31 (6%) |
| Within 6 steps of kitchen |  |  |  |  |  |  |  |
| Has water | 89 (9%) | 35 (7%) | 37 (7%) | 29 (5%) | 41 (8%) | 51 (10%) | 47 (9%) |
| Has soap | 27 (3%) | 12 (2%) | 11 (2%) | 7 (1%) | 11 (2%) | 20 (4%) | 15 (3%) |
| **Nutrition** |  |  |  |  |  |  |  |
| Food secure | 678 (68%) | 381 (72%) | 360 (69%) | 366 (69%) | 358 (68%) | 364 (68%) | 386 (71%) |

Data are n (%) or mean (standard deviation); No. of participants includes 21 sets of twins. This table presents data from participants re-enrolled with data collected on at least the first data collection day (day 1). Missing data for the following variables: Human feces observed in the house (6 missing), and child's play area (8 missing), Owned a potty (2 missing), Daily defection in the open for adult men (81 missing) and adult women (3 missing), Acres of agriculture land owned (199 missing), Paternal works in agriculture (2 missing), Maternal age (16 missing)

**Table D. Intra-cluster correlation by outcome**

|  | **Intervention arm** | | | | | | |
| --- | --- | --- | --- | --- | --- | --- | --- |
| **Outcomes** | **Control** | **Sanitation** | **Handwashing** | **Water** | **Nutrition** | **WSH** | **WSH+N** |
| **FSIQ** | 0.04 | 0.09 | 0.14 | 0.02 | 0.10 | 0.14 | 0.13 |
| **VCI** | 0.04 | 0.13 | 0.10 | ** | 0.06 | 0.10 | 0.09 |
| **CPI** | 0.05 | 0.07 | 0.09 | 0.03 | 0.07 | 0.12 | 0.14 |
| **FRI** | 0.04 | 0.02 | 0.13 | ** | 0.08 | 0.01 | 0.05 |
| **GAI** | 0.03 | 0.08 | 0.13 | 0.01 | 0.07 | 0.13 | 0.08 |
| **NVI** | 0.03 | 0.06 | 0.13 | 0.01 | 0.09 | 0.13 | 0.13 |
| **WMI** | 0.04 | 0.07 | 0.06 | 0.06 | 0.09 | 0.11 | 0.12 |
| **Fine motor** | ** | ** | ** | ** | ** | ** | ** |
| **Narrative memory** | 0.06 | 0.13 | 0.16 | 0.06 | 0.10 | 0.09 | 0.11 |
| **Forward word span** | 0.00 | 0.10 | 0.02 | ** | 0.01 | 0.08 | 0.03 |
| **Corsi blocks** | ** | ** | 0.03 | 0.02 | ** | 0.07 | 0.04 |
| **Spelling** | 0.06 | 0.08 | 0.18 | 0.05 | 0.11 | 0.11 | 0.12 |
| **Math** | 0.06 | ** | 0.09 | 0.05 | 0.07 | 0.12 | 0.11 |
| **Reading** | 0.07 | 0.07 | 0.17 | 0.03 | 0.08 | 0.13 | 0.13 |
| **SDQ prosocial** | 0.06 | 0.08 | 0.05 | 0.07 | 0.09 | 0.10 | 0.15 |
| **SDQ difficulty** | 0.09 | 0.11 | 0.09 | 0.17 | 0.10 | 0.12 | 0.10 |
| **HOME** | 0.12 | 0.29 | 0.24 | 0.27 | 0.24 | 0.28 | 0.31 |
| **CES-D** | 0.00 | ** | ** | ** | ** | 0.02 | 0.04 |

Intra-cluster correlations were estimated with linear mixed effect models with random effects at the block level. Internally standardized outcomes were used.

** indicates that the fitted model was singular.

FSIQ: Wechsler preschool and primary scales of intelligence Full Scale IQ; CPI: Cognitive Processing Index; VCI: Verbal Comprehension Index; FRI: Fluid reasoning index; GAI: General abilities index; NVI: Non-verbal index; WMI: Working memory index; Fine motor: Assessed using the Movement Assessment Battery for Children manual dexterity overall score; Narrative memory: Sum of free and cued recall scores from a narrative memory test; SDQ: Strengths and difficulties questionnaire; CES-D: Center for Epidemiologic Studies 20-question depression measure; HOME: Middle Childhood Home Observation Measurement of the Environment

**Table E. WPPSI results**

| **FSIQ z score** | **n** | **mean** | **unadj v C** | **adj v C** | **adj v N** | **adj v WSH** |
| --- | --- | --- | --- | --- | --- | --- |
| **Control** | 873 | 0.00 (1.00) | Ref | Ref | - | - |
| **Water** | 495 | 0.02 (1.03) | 0.01 (-0.09, 0.11) | 0.02 (-0.08, 0.13) | - | - |
| **Sanitation** | 484 | 0.16 (0.98) | 0.18 (0.05, 0.30) | 0.11 (-0.01, 0.22) | - | - |
| **Handwashing** | 492 | -0.02 (1.05) | -0.02 (-0.16, 0.12) | -0.02 (-0.15, 0.10) | - | - |
| **WSH** | 496 | 0.08 (0.98) | 0.07 (-0.06, 0.19) | 0.06 (-0.05, 0.17) | - | Ref |
| **Nutrition** | 494 | 0.05 (1.00) | 0.04 (-0.07, 0.15) | 0.07 (-0.03, 0.16) | Ref | - |
| **Nutrition+WSH** | 499 | 0.11 (1.00) | 0.12 (0.01, 0.22) | 0.12 (0.02, 0.23) | 0.05 (-0.09, 0.18) | 0.04 (-0.09, 0.17) |
| **CPI z score** | **n** | **mean** | **unadj v C** | **adj v C** | **adj v N** | **adj v WSH** |
| **Control** | 873 | 0.00 (1.00) | Ref | Ref | - | - |
| **Water** | 495 | -0.03 (1.02) | -0.03 (-0.14, 0.09) | -0.02 (-0.13, 0.09) | - | - |
| **Sanitation** | 484 | 0.07 (0.99) | 0.08 (-0.05, 0.20) | 0.03 (-0.09, 0.16) | - | - |
| **Handwashing** | 492 | -0.09 (1.05) | -0.10 (-0.23, 0.04) | -0.08 (-0.21, 0.05) | - | - |
| **WSH** | 496 | -0.02 (0.96) | -0.03 (-0.15, 0.09) | -0.04 (-0.14, 0.07) | - | Ref |
| **Nutrition** | 494 | -0.01 (0.99) | -0.02 (-0.13, 0.09) | 0.01 (-0.09, 0.11) | Ref | - |
| **Nutrition+WSH** | 499 | 0.08 (1.03) | 0.08 (-0.04, 0.20) | 0.09 (-0.02, 0.21) | 0.08 (-0.06, 0.22) | 0.11 (-0.02, 0.24) |
| **VCI z score** | **n** | **mean** | **unadj v C** | **adj v C** | **adj v N** | **adj v WSH** |
| **Control** | 873 | 0.00 (1.00) | Ref | Ref | - | - |
| **Water** | 495 | 0.08 (1.00) | 0.08 (-0.03, 0.18) | 0.07 (-0.03, 0.18) | - | - |
| **Sanitation** | 484 | 0.12 (1.01) | 0.14 (0.00, 0.27) | 0.07 (-0.05, 0.20) | - | - |
| **Handwashing** | 492 | 0.03 (1.06) | 0.03 (-0.11, 0.17) | 0.01 (-0.11, 0.14) | - | - |
| **WSH** | 496 | 0.05 (0.97) | 0.04 (-0.08, 0.17) | 0.03 (-0.09, 0.15) | - | Ref |
| **Nutrition** | 494 | 0.08 (1.01) | 0.07 (-0.04, 0.18) | 0.10 (0.00, 0.20) | Ref | - |
| **Nutrition+WSH** | 499 | 0.09 (0.98) | 0.10 (-0.01, 0.21) | 0.10 (-0.01, 0.22) | -0.01 (-0.15, 0.12) | 0.05 (-0.09, 0.18) |
| **FRI z score** | **n** | **mean** | **unadj v C** | **adj v C** | **adj v N** | **adj v WSH** |
| **Control** | 873 | 0.00 (1.00) | Ref | Ref | - | - |
| **Water** | 495 | 0.07 (1.01) | 0.07 (-0.04, 0.17) | 0.08 (-0.04, 0.19) | - | - |
| **Sanitation** | 484 | 0.16 (0.99) | 0.18 (0.06, 0.29) | 0.12 (0.00, 0.24) | - | - |
| **Handwashing** | 492 | -0.02 (1.02) | -0.02 (-0.16, 0.12) | -0.03 (-0.16, 0.10) | - | - |
| **WSH** | 496 | 0.10 (0.96) | 0.08 (-0.04, 0.19) | 0.10 (-0.01, 0.21) | - | Ref |
| **Nutrition** | 494 | 0.11 (1.05) | 0.10 (-0.01, 0.22) | 0.12 (0.01, 0.23) | Ref | - |
| **Nutrition+WSH** | 499 | 0.15 (0.98) | 0.16 (0.04, 0.28) | 0.15 (0.03, 0.27) | 0.02 (-0.10, 0.14) | 0.06 (-0.05, 0.17) |
| **GAI z score** | **n** | **mean** | **unadj v C** | **adj v C** | **adj v N** | **adj v WSH** |
| **Control** | 873 | 0.00 (1.00) | Ref | Ref | - | - |
| **Water** | 495 | 0.05 (1.01) | 0.04 (-0.06, 0.14) | 0.05 (-0.06, 0.15) | - | - |
| **Sanitation** | 484 | 0.17 (0.99) | 0.19 (0.07, 0.31) | 0.12 (0.01, 0.23) | - | - |
| **Handwashing** | 492 | -0.01 (1.04) | -0.01 (-0.15, 0.12) | -0.02 (-0.14, 0.10) | - | - |
| **WSH** | 496 | 0.11 (0.97) | 0.09 (-0.03, 0.21) | 0.08 (-0.03, 0.20) | - | Ref |
| **Nutrition** | 494 | 0.09 (0.99) | 0.07 (-0.04, 0.19) | 0.11 (0.01, 0.20) | Ref | - |
| **Nutrition+WSH** | 499 | 0.11 (0.97) | 0.12 (0.02, 0.22) | 0.12 (0.03, 0.22) | 0.01 (-0.13, 0.14) | 0.02 (-0.11, 0.14) |
| **NVI z score** | **n** | **mean** | **unadj v C** | **adj v C** | **adj v N** | **adj v WSH** |
| **Control** | 873 | 0.00 (1.00) | Ref | Ref | - | - |
| **Water** | 495 | 0.01 (1.02) | 0.00 (-0.10, 0.10) | 0.02 (-0.09, 0.12) | - | - |
| **Sanitation** | 484 | 0.16 (0.97) | 0.18 (0.06, 0.29) | 0.11 (0.00, 0.22) | - | - |
| **Handwashing** | 492 | -0.04 (1.04) | -0.04 (-0.17, 0.10) | -0.04 (-0.16, 0.08) | - | - |
| **WSH** | 496 | 0.09 (0.98) | 0.07 (-0.06, 0.19) | 0.07 (-0.04, 0.18) | - | Ref |
| **Nutrition** | 494 | 0.03 (1.02) | 0.02 (-0.08, 0.13) | 0.05 (-0.05, 0.14) | Ref | - |
| **Nutrition+WSH** | 499 | 0.12 (1.01) | 0.13 (0.02, 0.23) | 0.13 (0.03, 0.24) | 0.07 (-0.06, 0.21) | 0.05 (-0.09, 0.18) |
| **WMI z score** | **n** | **mean** | **unadj v C** | **adj v C** | **adj v N** | **adj v WSH** |
| **Control** | 873 | 0.00 (1.00) | Ref | Ref | - | - |
| **Water** | 495 | 0.01 (1.03) | 0.02 (-0.10, 0.14) | 0.02 (-0.10, 0.13) | - | - |
| **Sanitation** | 484 | 0.09 (1.03) | 0.10 (-0.04, 0.23) | 0.05 (-0.08, 0.18) | - | - |
| **Handwashing** | 492 | -0.09 (1.06) | -0.13 (-0.26, 0.01) | -0.09 (-0.22, 0.04) | - | - |
| **WSH** | 496 | 0.04 (0.97) | 0.02 (-0.11, 0.15) | 0.04 (-0.08, 0.15) | - | Ref |
| **Nutrition** | 494 | 0.05 (0.99) | 0.03 (-0.09, 0.15) | 0.05 (-0.06, 0.17) | Ref | - |
| **Nutrition+WSH** | 499 | 0.05 (1.02) | 0.02 (-0.10, 0.14) | 0.06 (-0.06, 0.17) | 0.00 (-0.16, 0.16) | 0.01 (-0.13, 0.14) |
| **FSIQ scaled score** | **n** | **mean** | **unadj v C** | **adj v C** | **adj v N** | **adj v WSH** |
| **Control** | 873 | 72.17 (8.10) | Ref | Ref | - | - |
| **Water** | 495 | 72.26 (8.68) | 0.07 (-0.73, 0.87) | 0.17 (-0.65, 0.99) | - | - |
| **Sanitation** | 484 | 73.32 (8.38) | 1.26 (0.26, 2.26) | 0.77 (-0.12, 1.65) | - | - |
| **Handwashing** | 492 | 72.08 (8.70) | -0.07 (-1.17, 1.04) | -0.08 (-1.06, 0.89) | - | - |
| **WSH** | 496 | 72.74 (8.31) | 0.51 (-0.51, 1.52) | 0.35 (-0.54, 1.24) | - | Ref |
| **Nutrition** | 494 | 72.71 (8.45) | 0.40 (-0.51, 1.32) | 0.58 (-0.21, 1.37) | Ref | - |
| **Nutrition+WSH** | 498 | 72.93 (8.12) | 0.78 (-0.10, 1.66) | 0.89 (0.04, 1.73) | 0.25 (-0.86, 1.37) | 0.31 (-0.75, 1.38) |

All outcomes except for the FSIQ scaled score are presented as internally standardized z-scores, and all results account for the clustered study design through Huber-White robust standard errors clustered at the block-level.

FSIQ: Wechsler preschool and primary scales of intelligence Full Scale IQ; CPI: Cognitive Processing Index; VCI: Verbal Comprehension Index; FRI: Fluid reasoning index; GAI: General abilities index; NVI: Non-verbal index; WMI: Working memory index; unadj v C represents the unadjusted mean difference between the specified intervention arm and the control arm; results specified with adj represents the mean difference between each intervention arm and either the control (adj v C), Nutrition (adj v N) or WSH (adj v WSH) from generalized linear models that adjust for child age, measurement period (pre-COVID or during COVID), and prognostic baseline control variables (significant at p<0.20 in a likelihood ratio test) to increase precision (for example of included control variables see Table G)

Note: Sample sizes for adjusted comparisons differ slightly from unadjusted comparisons. See Table F for sample sizes for adjusted analyses.

**Table F. WPPSI results: Adjusted analysis sample size**

| **Comparison** | **n** | **Intervention** | **n** |
| --- | --- | --- | --- |
| Control | 849 | Water | 490 |
| Control | 849 | Sanitation | 480 |
| Control | 849 | Handwashing | 488 |
| Control | 849 | WSH | 482 |
| Control | 840 | Nutrition | 486 |
| Control | 849 | Nutrition+WSH | 492 |
| Nutrition+WSH | 487 | Nutrition | 486 |
| Nutrition+WSH | 492 | WSH | 482 |

Sample size for each arm in each comparison made for WPPSI outcomes presented in table E, except FSIQ scaled score where Nutrition+WSH has one fewer observation for each contrast.

**Table G. Control variables included in adjusted regression models**

| Control Variable | FSIQ | VCI | CPI | FRI | GAI | NVI | WMI | Spelling | Math | Reading | Fine Motor | Narrative memory | FWS | Corsi blocks | SDQ difficulty | SDQ prosocial | CES-D | HOME |
| --- | --- | --- | --- | --- | --- | --- | --- | --- | --- | --- | --- | --- | --- | --- | --- | --- | --- | --- |
| Child sex | 0 | 0 | 0 | 0 | 0 | 0 | 0 | 0 | 0 | 0 | 0 | 0 | 0 | 0 | 0 | 0 | 0 | 1 |
| Mother age | 1 | 1 | 1 | 1 | 0 | 1 | 1 | 1 | 1 | 0 | 0 | 0 | 0 | 1 | 1 | 1 | 1 | 1 |
| Mother education | 1 | 1 | 1 | 1 | 1 | 1 | 1 | 1 | 1 | 1 | 1 | 1 | 1 | 1 | 1 | 1 | 1 | 1 |
| Mother height | 1 | 1 | 1 | 1 | 1 | 1 | 1 | 1 | 1 | 1 | 1 | 1 | 1 | 1 | 1 | 1 | 1 | 1 |
| Father education | 1 | 1 | 1 | 1 | 1 | 1 | 1 | 1 | 1 | 1 | 1 | 1 | 1 | 1 | 1 | 1 | 1 | 1 |
| Number of Children under 18 | 1 | 0 | 1 | 1 | 0 | 1 | 1 | 1 | 1 | 1 | 1 | 1 | 1 | 1 | 1 | 1 | 1 | 0 |
| Household size | 1 | 1 | 1 | 1 | 1 | 1 | 1 | 1 | 1 | 1 | 1 | 1 | 1 | 0 | 1 | 0 | 1 | 1 |
| Food insecurity | 1 | 1 | 1 | 1 | 1 | 1 | 1 | 1 | 1 | 1 | 1 | 1 | 1 | 1 | 1 | 1 | 1 | 1 |
| Concrete floor | 1 | 1 | 1 | 1 | 1 | 1 | 1 | 1 | 1 | 1 | 1 | 1 | 1 | 1 | 1 | 1 | 1 | 1 |
| Has bike | 1 | 1 | 1 | 1 | 1 | 1 | 1 | 1 | 1 | 1 | 1 | 1 | 1 | 1 | 1 | 1 | 1 | 1 |
| Has TV | 1 | 1 | 1 | 1 | 1 | 1 | 1 | 1 | 1 | 1 | 1 | 1 | 1 | 1 | 1 | 1 | 1 | 1 |
| Has wardrobe | 1 | 1 | 1 | 1 | 1 | 1 | 1 | 1 | 1 | 1 | 1 | 1 | 1 | 1 | 1 | 1 | 1 | 1 |
| Has table | 1 | 1 | 1 | 1 | 1 | 1 | 1 | 1 | 1 | 1 | 1 | 1 | 1 | 1 | 1 | 1 | 1 | 1 |
| Has chair | 1 | 1 | 1 | 1 | 1 | 1 | 1 | 1 | 1 | 1 | 1 | 1 | 1 | 1 | 1 | 1 | 1 | 1 |
| Has bed | 1 | 1 | 1 | 1 | 1 | 1 | 1 | 1 | 1 | 1 | 1 | 1 | 1 | 1 | 1 | 1 | 1 | 1 |
| Has stool | 1 | 0 | 1 | 1 | 1 | 0 | 1 | 1 | 0 | 0 | 0 | 0 | 0 | 0 | 1 | 1 | 0 | 0 |
| Has mobile phone | 1 | 1 | 1 | 1 | 1 | 1 | 1 | 1 | 1 | 1 | 1 | 1 | 1 | 1 | 1 | 1 | 1 | 1 |

This table presents the control variables selected for inclusion in the first contrast (sanitation vs control) for each outcome. 0 or 1 in each cell indicates if each control variable was included in analyses for each outcome, respectively. Control variables were included in the were associated with the outcome with p<0.20 in a likelihood ratio test.

FSIQ: Wechsler preschool and primary scales of intelligence Full Scale IQ; CPI: Cognitive Processing Index; VCI: Verbal Comprehension Index; FRI: Fluid reasoning index; GAI: General abilities index; NVI: Non-verbal index; WMI: Working memory index; Fine motor: Measured with the Movement assessment battery for children Manual Dexterity score; FWS: Forward word span; SDQ: Strengths and difficulties questionnaire; CES-D: Center for Epidemiologic Studies 20-question depression measure; HOME: Middle childhood Home Observation Measurement of the Environment

**Table H. Fine motor, Executive functioning, School achievement, SDQ results**

| **Fine motor MD** | **n** | **mean** | **unadj v C** | **adj v C** | **adj v N** | **adj v WSH** |
| --- | --- | --- | --- | --- | --- | --- |
| **Control** | **828** | 0.00 (1.00) | Ref | Ref | - | - |
| **Water** | **468** | -0.01 (0.95) | -0.11 (-0.31, 0.08) | -0.01 (-0.13, 0.12) | - | - |
| **Sanitation** | **451** | 0.05 (0.98) | 0.07 (-0.09, 0.23) | 0.05 (-0.07, 0.17) | - | - |
| **Handwashing** | **457** | 0.08 (1.05) | 0.13 (-0.10, 0.36) | 0.08 (-0.07, 0.23) | - | - |
| **WSH** | **459** | 0.06 (0.94) | 0.05 (-0.11, 0.20) | 0.08 (-0.04, 0.21) | - | Ref |
| **Nutrition** | **461** | 0.04 (0.91) | 0.10 (-0.10, 0.30) | 0.05 (-0.07, 0.17) | Ref | - |
| **Nutrition+WSH** | **468** | 0.04 (1.02) | 0.09 (-0.11, 0.28) | 0.06 (-0.07, 0.18) | -0.01 (-0.16, 0.13) | -0.02 (-0.16, 0.12) |
| **Fine motor MD1** | **n** | **mean** | **unadj v C** | **adj v C** | **adj v N** | **adj v WSH** |
| **Control** | **864** | -0.01 (1.01) | Ref | Ref | - | - |
| **Water** | **493** | -0.06 (1.00) | -0.07 (-0.21, 0.06) | -0.04 (-0.17, 0.08) | - | - |
| **Sanitation** | **483** | 0.01 (1.00) | 0.01 (-0.11, 0.13) | 0.01 (-0.11, 0.12) | - | - |
| **Handwashing** | **486** | 0.04 (0.86) | 0.04 (-0.09, 0.18) | 0.04 (-0.09, 0.17) | - | - |
| **WSH** | **493** | -0.04 (1.07) | -0.05 (-0.18, 0.09) | -0.04 (-0.17, 0.10) | - | Ref |
| **Nutrition** | **490** | -0.06 (1.17) | -0.04 (-0.19, 0.10) | -0.05 (-0.19, 0.09) | Ref | - |
| **Nutrition+WSH** | **495** | 0.04 (0.96) | 0.05 (-0.08, 0.18) | 0.06 (-0.07, 0.19) | 0.10 (-0.05, 0.24) | 0.11 (-0.02, 0.24) |
| **Fine motor MD2** | **n** | **mean** | **unadj v C** | **adj v C** | **adj v N** | **adj v WSH** |
| **Control** | **849** | -0.01 (1.00) | Ref | Ref | - | - |
| **Water** | **482** | -0.05 (1.22) | -0.04 (-0.20, 0.13) | -0.02 (-0.16, 0.13) | - | - |
| **Sanitation** | **466** | 0.01 (0.97) | 0.02 (-0.12, 0.17) | 0.03 (-0.11, 0.17) | - | - |
| **Handwashing** | **475** | 0.00 (1.34) | 0.07 (-0.13, 0.27) | 0.03 (-0.13, 0.18) | - | - |
| **WSH** | **472** | 0.01 (0.95) | 0.07 (-0.08, 0.23) | 0.05 (-0.08, 0.17) | - | Ref |
| **Nutrition** | **478** | -0.03 (1.01) | -0.03 (-0.18, 0.12) | 0.01 (-0.11, 0.12) | Ref | - |
| **Nutrition+WSH** | **477** | 0.03 (1.07) | 0.00 (-0.19, 0.18) | 0.06 (-0.06, 0.18) | 0.03 (-0.14, 0.20) | 0.00 (-0.14, 0.15) |
| **Fine motor MD3** | **n** | **mean** | **unadj v C** | **adj v C** | **adj v N** | **adj v WSH** |
| **Control** | **854** | 0.00 (1.02) | Ref | Ref | - | - |
| **Water** | **477** | -0.04 (1.07) | -0.07 (-0.22, 0.09) | -0.03 (-0.14, 0.09) | - | - |
| **Sanitation** | **469** | 0.02 (1.24) | 0.07 (-0.08, 0.21) | 0.02 (-0.11, 0.14) | - | - |
| **Handwashing** | **475** | 0.03 (1.08) | 0.12 (-0.05, 0.29) | 0.05 (-0.09, 0.18) | - | - |
| **WSH** | **479** | 0.09 (0.94) | 0.04 (-0.12, 0.19) | 0.11 (-0.01, 0.24) | - | Ref |
| **Nutrition** | **473** | 0.06 (0.93) | 0.10 (-0.05, 0.26) | 0.09 (-0.03, 0.20) | Ref | - |
| **Nutrition+WSH** | **488** | -0.03 (1.18) | 0.02 (-0.14, 0.18) | -0.02 (-0.15, 0.11) | -0.10 (-0.26, 0.05) | -0.11 (-0.26, 0.04) |
| **Narrative memory** | **n** | **mean** | **unadj v C** | **adj v C** | **adj v N** | **adj v WSH** |
| **Control** | **873** | -0.01 (1.00) | Ref | Ref | - | - |
| **Water** | **495** | 0.04 (1.07) | 0.04 (-0.08, 0.16) | 0.05 (-0.08, 0.17) | - | - |
| **Sanitation** | **484** | 0.10 (1.08) | 0.11 (-0.03, 0.25) | 0.06 (-0.07, 0.19) | - | - |
| **Handwashing** | **492** | 0.02 (1.05) | 0.06 (-0.08, 0.19) | 0.04 (-0.09, 0.16) | - | - |
| **WSH** | **496** | -0.03 (0.98) | 0.00 (-0.12, 0.12) | -0.04 (-0.16, 0.07) | - | Ref |
| **Nutrition** | **494** | 0.07 (0.98) | 0.07 (-0.04, 0.18) | 0.08 (-0.03, 0.19) | Ref | - |
| **Nutrition+WSH** | **499** | 0.08 (0.99) | 0.10 (-0.01, 0.22) | 0.10 (-0.01, 0.21) | 0.03 (-0.11, 0.18) | 0.12 (-0.02, 0.25) |
| **Forward word span** | **n** | **mean** | **unadj v C** | **adj v C** | **adj v N** | **adj v WSH** |
| **Control** | **873** | 0.00 (1.00) | Ref | Ref | - | - |
| **Water** | **495** | 0.08 (1.09) | 0.08 (-0.04, 0.20) | 0.09 (-0.05, 0.22) | - | - |
| **Sanitation** | **484** | 0.10 (1.04) | 0.12 (0.00, 0.23) | 0.07 (-0.04, 0.19) | - | - |
| **Handwashing** | **492** | -0.02 (1.01) | -0.04 (-0.16, 0.07) | -0.04 (-0.16, 0.08) | - | - |
| **WSH** | **496** | 0.09 (1.05) | 0.08 (-0.05, 0.21) | 0.05 (-0.08, 0.17) | - | Ref |
| **Nutrition** | **494** | 0.05 (0.98) | 0.03 (-0.08, 0.14) | 0.03 (-0.07, 0.14) | Ref | - |
| **Nutrition+WSH** | **499** | 0.02 (1.04) | 0.03 (-0.07, 0.13) | 0.02 (-0.08, 0.13) | -0.01 (-0.15, 0.12) | -0.03 (-0.18, 0.13) |
| **Corsi blocks** | **n** | **mean** | **unadj v C** | **adj v C** | **adj v N** | **adj v WSH** |
| **Control** | **873** | 0.00 (1.00) | Ref | Ref | - | - |
| **Water** | **495** | -0.02 (1.05) | 0.02 (-0.10, 0.13) | 0.01 (-0.11, 0.13) | - | - |
| **Sanitation** | **484** | 0.09 (0.98) | 0.11 (0.00, 0.21) | 0.09 (-0.02, 0.19) | - | - |
| **Handwashing** | **492** | 0.00 (1.01) | 0.01 (-0.11, 0.13) | 0.02 (-0.10, 0.15) | - | - |
| **WSH** | **496** | 0.01 (1.02) | 0.03 (-0.08, 0.15) | 0.01 (-0.09, 0.12) | - | Ref |
| **Nutrition** | **494** | 0.02 (0.95) | 0.02 (-0.08, 0.12) | 0.03 (-0.06, 0.12) | Ref | - |
| **Nutrition+WSH** | **499** | 0.03 (0.98) | 0.06 (-0.05, 0.16) | 0.04 (-0.06, 0.15) | 0.00 (-0.13, 0.13) | 0.04 (-0.09, 0.18) |
| **Spelling** | **n** | **mean** | **unadj v C** | **adj v C** | **adj v N** | **adj v WSH** |
| **Control** | **873** | 0.00 (1.00) | Ref | Ref | - | - |
| **Water** | **495** | -0.01 (1.02) | -0.01 (-0.13, 0.10) | 0.00 (-0.11, 0.11) | - | - |
| **Sanitation** | **484** | 0.06 (0.99) | 0.06 (-0.06, 0.18) | 0.01 (-0.10, 0.13) | - | - |
| **Handwashing** | **492** | -0.03 (1.04) | -0.05 (-0.19, 0.08) | -0.04 (-0.15, 0.08) | - | - |
| **WSH** | **496** | 0.02 (1.03) | 0.00 (-0.13, 0.13) | 0.00 (-0.11, 0.11) | - | Ref |
| **Nutrition** | **494** | 0.04 (1.04) | 0.04 (-0.08, 0.17) | 0.03 (-0.09, 0.15) | Ref | - |
| **Nutrition+WSH** | **499** | 0.03 (1.00) | 0.02 (-0.10, 0.13) | 0.03 (-0.08, 0.14) | -0.02 (-0.14, 0.11) | 0.03 (-0.10, 0.16) |
| **Math** | **n** | **mean** | **unadj v C** | **adj v C** | **adj v N** | **adj v WSH** |
| **Control** | **873** | 0.00 (1.01) | Ref | Ref | - | - |
| **Water** | **495** | 0.02 (1.06) | 0.00 (-0.12, 0.12) | 0.03 (-0.09, 0.15) | - | - |
| **Sanitation** | **484** | 0.11 (1.03) | 0.10 (-0.02, 0.22) | 0.06 (-0.06, 0.18) | - | - |
| **Handwashing** | **492** | -0.01 (1.05) | -0.03 (-0.16, 0.09) | -0.02 (-0.13, 0.09) | - | - |
| **WSH** | **496** | 0.05 (1.02) | 0.05 (-0.08, 0.18) | 0.03 (-0.09, 0.15) | - | Ref |
| **Nutrition** | **494** | 0.05 (1.03) | 0.05 (-0.08, 0.17) | 0.04 (-0.08, 0.16) | Ref | - |
| **Nutrition+WSH** | **499** | 0.07 (1.03) | 0.07 (-0.06, 0.19) | 0.08 (-0.04, 0.20) | 0.03 (-0.11, 0.16) | 0.05 (-0.09, 0.19) |
| **Read** | **n** | **mean** | **unadj v C** | **adj v C** | **adj v N** | **adj v WSH** |
| **Control** | **873** | 0.01 (1.01) | Ref | Ref | - | - |
| **Water** | **495** | -0.01 (1.01) | -0.02 (-0.13, 0.09) | 0.00 (-0.11, 0.11) | - | - |
| **Sanitation** | **484** | 0.02 (0.97) | 0.03 (-0.09, 0.15) | -0.02 (-0.14, 0.10) | - | - |
| **Handwashing** | **492** | -0.03 (1.04) | -0.06 (-0.19, 0.07) | -0.04 (-0.16, 0.08) | - | - |
| **WSH** | **496** | 0.03 (1.05) | -0.01 (-0.15, 0.14) | 0.01 (-0.11, 0.13) | - | Ref |
| **Nutrition** | **494** | 0.02 (1.01) | 0.02 (-0.10, 0.15) | 0.01 (-0.11, 0.13) | Ref | - |
| **Nutrition+WSH** | **499** | 0.02 (1.00) | 0.01 (-0.12, 0.14) | 0.02 (-0.10, 0.14) | -0.01 (-0.13, 0.12) | 0.02 (-0.12, 0.16) |
| **SDQ difficulty** | **n** | **mean** | **unadj v C** | **adj v C** | **adj v N** | **adj v WSH** |
| **Control** | **983** | -0.01 (0.99) | Ref | Ref | - | - |
| **Water** | **527** | -0.17 (0.98) | -0.18 (-0.33,-0.03) | -0.17 (-0.32,-0.02) | - | - |
| **Sanitation** | **515** | -0.08 (0.93) | -0.07 (-0.21, 0.06) | -0.07 (-0.20, 0.06) | - | - |
| **Handwashing** | **527** | -0.18 (0.94) | -0.15 (-0.28,-0.02) | -0.16 (-0.28,-0.03) | - | - |
| **WSH** | **519** | -0.29 (1.06) | -0.28 (-0.43,-0.14) | -0.31 (-0.45,-0.17) | - | Ref |
| **Nutrition** | **528** | -0.21 (0.91) | -0.23 (-0.38,-0.09) | -0.22 (-0.35,-0.09) | Ref | - |
| **Nutrition+WSH** | **534** | -0.17 (0.92) | -0.13 (-0.27, 0.01) | -0.15 (-0.28,-0.01) | 0.08 (-0.05, 0.22) | 0.17 (0.03, 0.32) |
| **SDQ prosocial** | **n** | **mean** | **unadj v C** | **adj v C** | **adj v N** | **adj v WSH** |
| **Control** | **983** | 0.02 (1.00) | Ref | Ref | - | - |
| **Water** | **527** | 0.19 (0.97) | 0.19 (0.06, 0.32) | 0.20 (0.07, 0.33) | - | - |
| **Sanitation** | **515** | 0.12 (1.03) | 0.11 (-0.02, 0.24) | 0.09 (-0.04, 0.23) | - | - |
| **Handwashing** | **527** | 0.22 (0.93) | 0.21 (0.08, 0.35) | 0.22 (0.09, 0.34) | - | - |
| **WSH** | **519** | 0.29 (0.97) | 0.28 (0.13, 0.42) | 0.28 (0.15, 0.42) | - | Ref |
| **Nutrition** | **528** | 0.20 (0.97) | 0.21 (0.08, 0.34) | 0.21 (0.07, 0.34) | Ref | - |
| **Nutrition+WSH** | **534** | 0.33 (1.00) | 0.31 (0.15, 0.46) | 0.31 (0.16, 0.46) | 0.10 (-0.06, 0.27) | 0.02 (-0.14, 0.17) |

All outcomes are presented as internally standardized z-scores, and all results account for the clustered study design through Huber-White robust standard errors clustered at the block-level. Sample size for adjusted comparisons differ slightly from unadjusted comparisons. See Table I for sample size for adjusted analyses. Fine motor individual tests raw scores exclude children who failed on the assessment, the composite manual dexterity scores excluded children who failed on one or more assessment.

Fine motor: Measured with the Movement assessment battery for children Manual Dexterity (MD) score; MD: manual dexterity overall score (created by summing internally standardized scores on MD1, MD2, and MD3 and then re-standardizing the sum score); MD1: first measure of manual dexterity – posting coins or placing pegs (depending on child age); MD2: second measure of manual dexterity – Threading lace; MD3: third measure of manual dexterity – Drawing trails; SDQ: Strengths and difficulties questionnaire; Narrative memory: Sum of free and cued recall scores from a narrative memory test; SDQ: Strengths and difficulties questionnaire; unadj v C represents the unadjusted mean difference between the specified intervention arm and the control arm; results specified with adj represents the mean difference between each intervention arm and either the control (adj v C), Nutrition (adj v N) or WSH (adj v WSH) from generalized linear models that adjust for child age, measurement period (pre-COVID or during COVID), and prognostic baseline control variables (significant at p<0.20 in a likelihood ratio test) to increase precision (for example of included control variables see Table G)

**Table I. Fine motor, Executive functioning, School achievement, SDQ results: adjusted analysis sample size**

| **Fine motor MD** | |  |  |
| --- | --- | --- | --- |
| **Comparison** | **n** | **Intervention** | **n** |
| Control | 805 | Water | 463 |
| Control | 805 | Sanitation | 447 |
| Control | 805 | Handwashing | 453 |
| Control | 805 | WSH | 445 |
| Control | 796 | Nutrition | 454 |
| Control | 805 | Nutrition+WSH | 462 |
| Nutrition+WSH | 457 | Nutrition | 454 |
| Nutrition+WSH | 462 | WSH | 445 |
| **Fine motor MD1** | | |  |
| **Comparison** | **n** | **Intervention** | **n** |
| Control | 840 | Water | 488 |
| Control | 840 | Sanitation | 479 |
| Control | 840 | Handwashing | 482 |
| Control | 840 | WSH | 479 |
| Control | 831 | Nutrition | 482 |
| Control | 840 | Nutrition+WSH | 488 |
| Nutrition+WSH | 483 | Nutrition | 482 |
| Nutrition+WSH | 488 | WSH | 479 |
| **Fine motor MD2** | | |  |
| **Comparison** | **n** | **Intervention** | **n** |
| Control | 825 | Water | 477 |
| Control | 825 | Sanitation | 462 |
| Control | 825 | Handwashing | 471 |
| Control | 825 | WSH | 458 |
| Control | 816 | Nutrition | 470 |
| Control | 825 | Nutrition+WSH | 471 |
| Nutrition+WSH | 466 | Nutrition | 470 |
| Nutrition+WSH | 471 | WSH | 458 |
| **Fine motor MD3** | | |  |
| **Comparison** | **n** | **Intervention** | **n** |
| Control | 831 | Water | 472 |
| Control | 831 | Sanitation | 465 |
| Control | 831 | Handwashing | 471 |
| Control | 831 | WSH | 465 |
| Control | 822 | Nutrition | 466 |
| Control | 831 | Nutrition+WSH | 481 |
| Nutrition+WSH | 476 | Nutrition | 466 |
| Nutrition+WSH | 481 | WSH | 465 |
| **Narrative memory** | | |  |
| **Comparison** | **n** | **Intervention** | **n** |
| Control | 849 | Water | 490 |
| Control | 849 | Sanitation | 480 |
| Control | 849 | Handwashing | 488 |
| Control | 849 | WSH | 482 |
| Control | 840 | Nutrition | 486 |
| Control | 849 | Nutrition+WSH | 492 |
| Nutrition+WSH | 487 | Nutrition | 486 |
| Nutrition+WSH | 492 | WSH | 482 |
| **Forward word span** | | |  |
| **Comparison** | **n** | **Intervention** | **n** |
| Control | 849 | Water | 490 |
| Control | 849 | Sanitation | 480 |
| Control | 849 | Handwashing | 488 |
| Control | 849 | WSH | 482 |
| Control | 840 | Nutrition | 486 |
| Control | 849 | Nutrition+WSH | 492 |
| Nutrition+WSH | 487 | Nutrition | 486 |
| Nutrition+WSH | 492 | WSH | 482 |
| **Corsi blocks** | | |  |
| **Comparison** | **n** | **Intervention** | **n** |
| Control | 849 | Water | 490 |
| Control | 849 | Sanitation | 480 |
| Control | 849 | Handwashing | 488 |
| Control | 849 | WSH | 482 |
| Control | 840 | Nutrition | 486 |
| Control | 849 | Nutrition+WSH | 492 |
| Nutrition+WSH | 487 | Nutrition | 486 |
| Nutrition+WSH | 492 | WSH | 482 |
| **Spelling** | |  |  |
| **Comparison** | **n** | **Intervention** | **n** |
| Control | 849 | Water | 490 |
| Control | 849 | Sanitation | 480 |
| Control | 849 | Handwashing | 488 |
| Control | 849 | WSH | 482 |
| Control | 840 | Nutrition | 486 |
| Control | 849 | Nutrition+WSH | 492 |
| Nutrition+WSH | 487 | Nutrition | 486 |
| Nutrition+WSH | 492 | WSH | 482 |
| **Math** | | |  |
| **Comparison** | **n** | **Intervention** | **n** |
| Control | 849 | Water | 490 |
| Control | 849 | Sanitation | 480 |
| Control | 849 | Handwashing | 488 |
| Control | 849 | WSH | 482 |
| Control | 840 | Nutrition | 486 |
| Control | 849 | Nutrition+WSH | 492 |
| Nutrition+WSH | 487 | Nutrition | 486 |
| Nutrition+WSH | 492 | WSH | 482 |
| **Read** | | |  |
| **Comparison** | **n** | **Intervention** | **n** |
| Control | 849 | Water | 490 |
| Control | 849 | Sanitation | 480 |
| Control | 849 | Handwashing | 488 |
| Control | 849 | WSH | 482 |
| Control | 840 | Nutrition | 486 |
| Control | 849 | Nutrition+WSH | 492 |
| Nutrition+WSH | 487 | Nutrition | 486 |
| Nutrition+WSH | 492 | WSH | 482 |
| **SDQ difficulty** | | |  |
| **Comparison** | **n** | **Intervention** | **n** |
| Control | 955 | Water | 522 |
| Control | 955 | Sanitation | 510 |
| Control | 955 | Handwashing | 522 |
| Control | 955 | WSH | 503 |
| Control | 955 | Nutrition | 519 |
| Control | 955 | Nutrition+WSH | 527 |
| Nutrition+WSH | 527 | Nutrition | 519 |
| Nutrition+WSH | 527 | WSH | 503 |
| **SDQ prosocial** | | |  |
| **Comparison** | **n** | **Intervention** | **n** |
| Control | 955 | Water | 522 |
| Control | 955 | Sanitation | 510 |
| Control | 955 | Handwashing | 522 |
| Control | 955 | WSH | 503 |
| Control | 955 | Nutrition | 519 |
| Control | 955 | Nutrition+WSH | 527 |
| Nutrition+WSH | 527 | Nutrition | 519 |
| Nutrition+WSH | 527 | WSH | 503 |

This table presents the sample size for each arm in each comparison made for results presented in Table H

**Table J. SDQ difficulties subscale results**

| **SDQ Conduct** | **n** | **mean** | **unadj v C** | **adj v C** | **adj v N** | **adj v WSH** |
| --- | --- | --- | --- | --- | --- | --- |
| **Control** | **983** | -0.01 (0.99) | Ref | Ref | - | - |
| **Water** | **527** | -0.13 (1.00) | -0.14 (-0.27,-0.02) | -0.13 (-0.25, 0.00) | - | - |
| **Sanitation** | **515** | -0.06 (0.95) | -0.05 (-0.16, 0.07) | -0.04 (-0.16, 0.07) | - | - |
| **Handwashing** | **527** | -0.12 (0.95) | -0.10 (-0.22, 0.02) | -0.11 (-0.23, 0.01) | - | - |
| **WSH** | **519** | -0.23 (1.01) | -0.22 (-0.37,-0.08) | -0.24 (-0.38,-0.10) | - | Ref |
| **Nutrition** | **528** | -0.16 (0.96) | -0.16 (-0.30,-0.03) | -0.17 (-0.29,-0.04) | Ref | - |
| **Nutrition+WSH** | **534** | -0.11 (0.93) | -0.08 (-0.21, 0.04) | -0.10 (-0.23, 0.03) | 0.08 (-0.04, 0.20) | 0.15 (0.01, 0.29) |
| **SDQ Peer** | **n** | **mean** | **unadj v C** | **adj v C** | **adj v N** | **adj v WSH** |
| **Control** | **983** | -0.01 (0.99) | Ref | Ref | - | - |
| **Water** | **527** | -0.05 (0.98) | -0.05 (-0.21, 0.11) | -0.06 (-0.21, 0.10) | - | - |
| **Sanitation** | **515** | -0.01 (1.01) | 0.03 (-0.11, 0.17) | -0.02 (-0.16, 0.12) | - | - |
| **Handwashing** | **527** | -0.01 (1.02) | -0.01 (-0.18, 0.16) | 0.00 (-0.17, 0.16) | - | - |
| **WSH** | **519** | -0.12 (0.94) | -0.11 (-0.25, 0.02) | -0.13 (-0.26, 0.01) | - | Ref |
| **Nutrition** | **528** | -0.03 (0.91) | -0.07 (-0.21, 0.08) | -0.03 (-0.17, 0.12) | Ref | - |
| **Nutrition+WSH** | **534** | -0.08 (0.96) | -0.08 (-0.24, 0.08) | -0.08 (-0.24, 0.08) | -0.01 (-0.16, 0.14) | 0.07 (-0.10, 0.23) |
| **SDQ Hyperactive** | **n** | **mean** | **unadj v C** | **adj v C** | **adj v N** | **adj v WSH** |
| **Control** | **983** | 0.00 (1.00) | Ref | Ref | - | - |
| **Water** | **527** | -0.13 (0.97) | -0.13 (-0.26, 0.01) | -0.13 (-0.27, 0.01) | - | - |
| **Sanitation** | **515** | -0.04 (0.94) | -0.05 (-0.18, 0.08) | -0.04 (-0.17, 0.09) | - | - |
| **Handwashing** | **527** | -0.16 (0.97) | -0.17 (-0.29,-0.04) | -0.14 (-0.26,-0.02) | - | - |
| **WSH** | **519** | -0.21 (1.04) | -0.22 (-0.35,-0.08) | -0.22 (-0.36,-0.09) | - | Ref |
| **Nutrition** | **528** | -0.10 (0.94) | -0.12 (-0.24, 0.01) | -0.10 (-0.22, 0.02) | Ref | - |
| **Nutrition+WSH** | **534** | -0.12 (0.92) | -0.09 (-0.21, 0.03) | -0.10 (-0.22, 0.02) | 0.01 (-0.11, 0.13) | 0.13 (-0.02, 0.28) |
| **SDQ Emotion** | **n** | **mean** | **unadj v C** | **adj v C** | **adj v N** | **adj v WSH** |
| **Control** | **983** | -0.02 (0.99) | Ref | Ref | - | - |
| **Water** | **527** | -0.11 (0.96) | -0.11 (-0.24, 0.01) | -0.10 (-0.23, 0.02) | - | - |
| **Sanitation** | **515** | -0.09 (0.94) | -0.09 (-0.23, 0.04) | -0.07 (-0.21, 0.06) | - | - |
| **Handwashing** | **527** | -0.12 (0.99) | -0.08 (-0.20, 0.05) | -0.11 (-0.23, 0.01) | - | - |
| **WSH** | **519** | -0.15 (1.01) | -0.13 (-0.26, 0.00) | -0.16 (-0.29,-0.03) | - | Ref |
| **Nutrition** | **528** | -0.24 (0.91) | -0.24 (-0.38,-0.10) | -0.24 (-0.38,-0.11) | Ref | - |
| **Nutrition+WSH** | **534** | -0.09 (1.00) | -0.07 (-0.19, 0.06) | -0.07 (-0.20, 0.05) | 0.14 (-0.01, 0.29) | 0.07 (-0.09, 0.23) |

SDQ: Strengths and difficulties questionnaire; unadj v C represents the unadjusted mean difference between the specified intervention arm and the control arm; results specified with adj represents the mean difference between each intervention arm and either the control (adj v C), Nutrition (adj v N) or WSH (adj v WSH) from generalized linear models that adjust for child age, measurement period (pre-COVID or during COVID), and prognostic baseline control variables (significant at p<0.20 in a likelihood ratio test) to increase precision.

**Table K. Secondary outcome results**

| **CES-D** | **n** | **mean** | **unadj v C** | **adj v C** | **adj v N** | **adj v WSH** |
| --- | --- | --- | --- | --- | --- | --- |
| **Control** | 865 | 0.00 (1.00) | Ref | Ref | - | - |
| **Water** | 495 | 0.04 (1.06) | 0.01 (-0.11, 0.13) | 0.05 (-0.06, 0.16) | - | - |
| **Sanitation** | 483 | -0.06 (1.05) | -0.07 (-0.19, 0.05) | -0.05 (-0.17, 0.06) | - | - |
| **Handwashing** | 486 | -0.15 (0.98) | -0.18 (-0.30, -0.07) | -0.14 (-0.24, -0.03) | - | - |
| **WSH** | 496 | -0.04 (1.04) | -0.03 (-0.16, 0.10) | -0.03 (-0.16, 0.10) | - | Ref |
| **Nutrition** | 491 | -0.22 (0.93) | -0.23 (-0.34, -0.12) | -0.21 (-0.31, -0.11) | Ref | - |
| **Nutrition+WSH** | 496 | -0.14 (1.02) | -0.13 (-0.27, 0.01) | -0.15 (-0.27, -0.02) | 0.11 (-0.02, 0.24) | -0.09 (-0.24, 0.06) |
| **HOME** | **n** | **mean** | **unadj v C** | **adj v C** | **adj v N** | **adj v WSH** |
| **Control** | 986 | 0.01 (0.99) | Ref | Ref | - | - |
| **Water** | 528 | 0.21 (1.00) | 0.21 (0.05, 0.37) | 0.21 (0.05, 0.36) | - | - |
| **Sanitation** | 516 | 0.20 (0.98) | 0.23 (0.08, 0.39) | 0.17 (0.01, 0.33) | - | - |
| **Handwashing** | 528 | 0.23 (0.93) | 0.24 (0.09, 0.40) | 0.26 (0.11, 0.40) | - | - |
| **WSH** | 522 | 0.38 (0.98) | 0.35 (0.20, 0.50) | 0.35 (0.20, 0.51) | - | Ref |
| **Nutrition** | 529 | 0.26 (1.01) | 0.27 (0.11, 0.43) | 0.27 (0.12, 0.42) | Ref | - |
| **Nutrition+WSH** | 534 | 0.40 (0.95) | 0.42 (0.26, 0.58) | 0.40 (0.25, 0.56) | 0.15 (-0.04, 0.34) | 0.04 (-0.15, 0.22) |

All outcomes are presented as internally standardized z-scores, and all results account for the clustered study design through Huber-White robust standard errors clustered at the block-level. See Table M for sample size for adjusted analyses. CES-D: Center for Epidemiologic Studies 20-question depression measure; HOME: Middle childhood Home Observation Measurement of the Environment; unadj v C represents the unadjusted mean difference between the specified intervention arm and the control arm; results specified with adj represents the mean difference between each intervention arm and either the control (adj v C), Nutrition (adj v N) or WSH (adj v WSH) from generalized linear models that adjust for child age, measurement period (pre-COVID or during COVID), and prognostic baseline control variables (significant at p<0.20 in a likelihood ratio test) to increase precision (for example of included control variables see Table G)

**Table L. Secondary outcome results: adjusted analysis n’s**

| **CES-D** | |  |  |
| --- | --- | --- | --- |
| **Comparison** | **n** | **Intervention** | **n** |
| Control | 842 | Water | 490 |
| Control | 842 | Sanitation | 479 |
| Control | 842 | Handwashing | 482 |
| Control | 842 | WSH | 482 |
| Control | 833 | Nutrition | 483 |
| Control | 842 | Nutrition+WSH | 489 |
| Nutrition+WSH | 484 | Nutrition | 483 |
| Nutrition+WSH | 489 | WSH | 482 |
| **HOME** | |  |  |
| **Comparison** | **n** | **Intervention** | **n** |
| Control | 955 | Water | 522 |
| Control | 955 | Sanitation | 510 |
| Control | 955 | Handwashing | 522 |
| Control | 955 | WSH | 504 |
| Control | 955 | Nutrition | 519 |
| Control | 955 | Nutrition+WSH | 527 |
| Nutrition+WSH | 527 | Nutrition | 519 |
| Nutrition+WSH | 527 | WSH | 504 |

n’s for each arm in each comparison made for results presented in Table K

**Figure A. WPPSI index scores
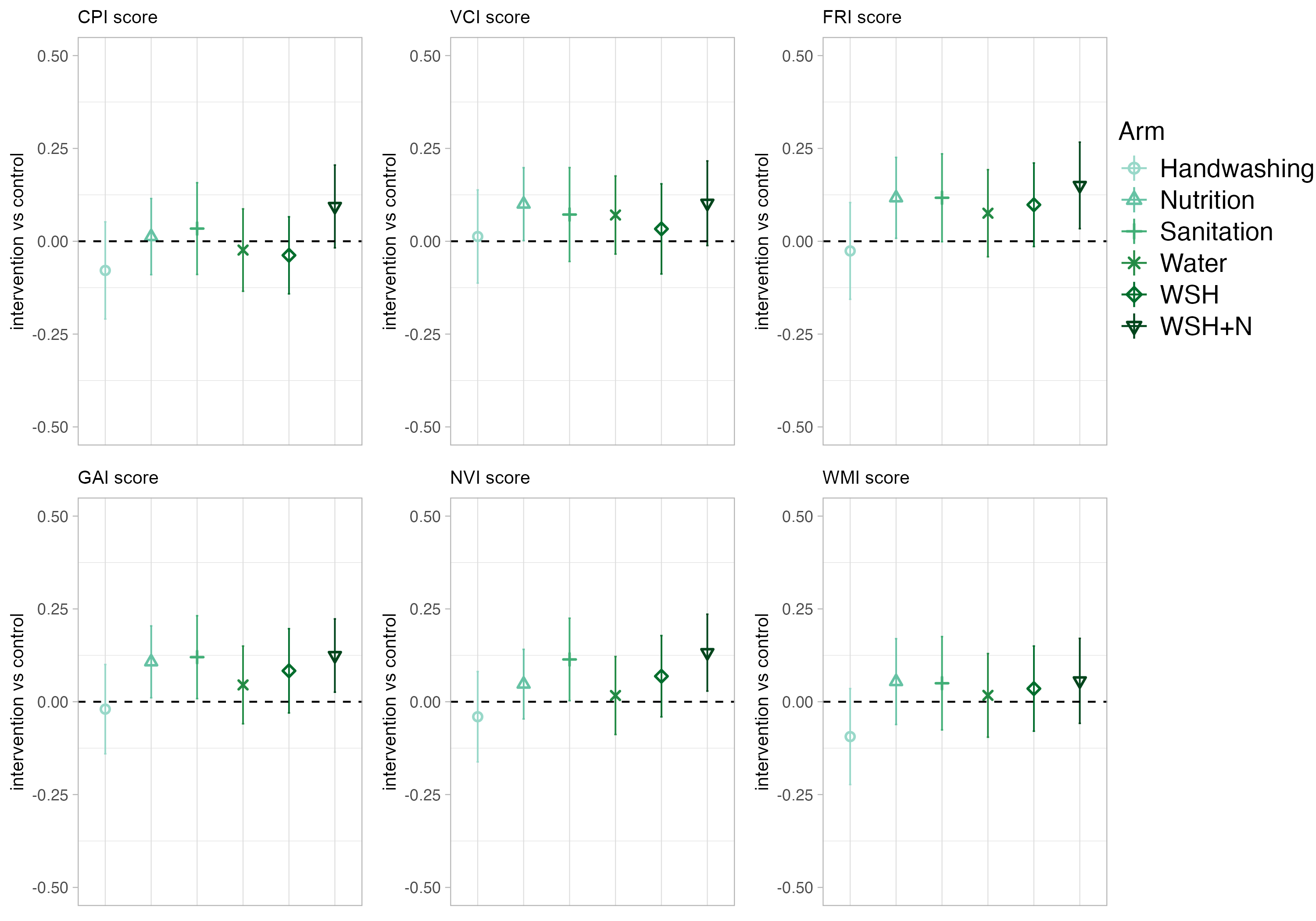
**

Point estimates reflect the mean difference between each intervention arm and the control arm from generalized linear models that adjust for child age, measurement period (pre-COVID or during COVID), and prognostic baseline control variables (significant at p<0.20 in a likelihood ratio test) to increase precision (for example of included control variables see Table G). All outcomes are internally standardized z-scores, and all results account for the clustered study design through Huber-White robust standard errors clustered at the block-level. CPI: Cognitive Processing Index; VCI: Verbal Comprehension Index; FRI: Fluid reasoning index; GAI: General abilities index; NVI: Non-verbal index; WMI: Working memory index. Bars represent 95% confidence intervals.

**Figure B. Expanded narrative memory results**


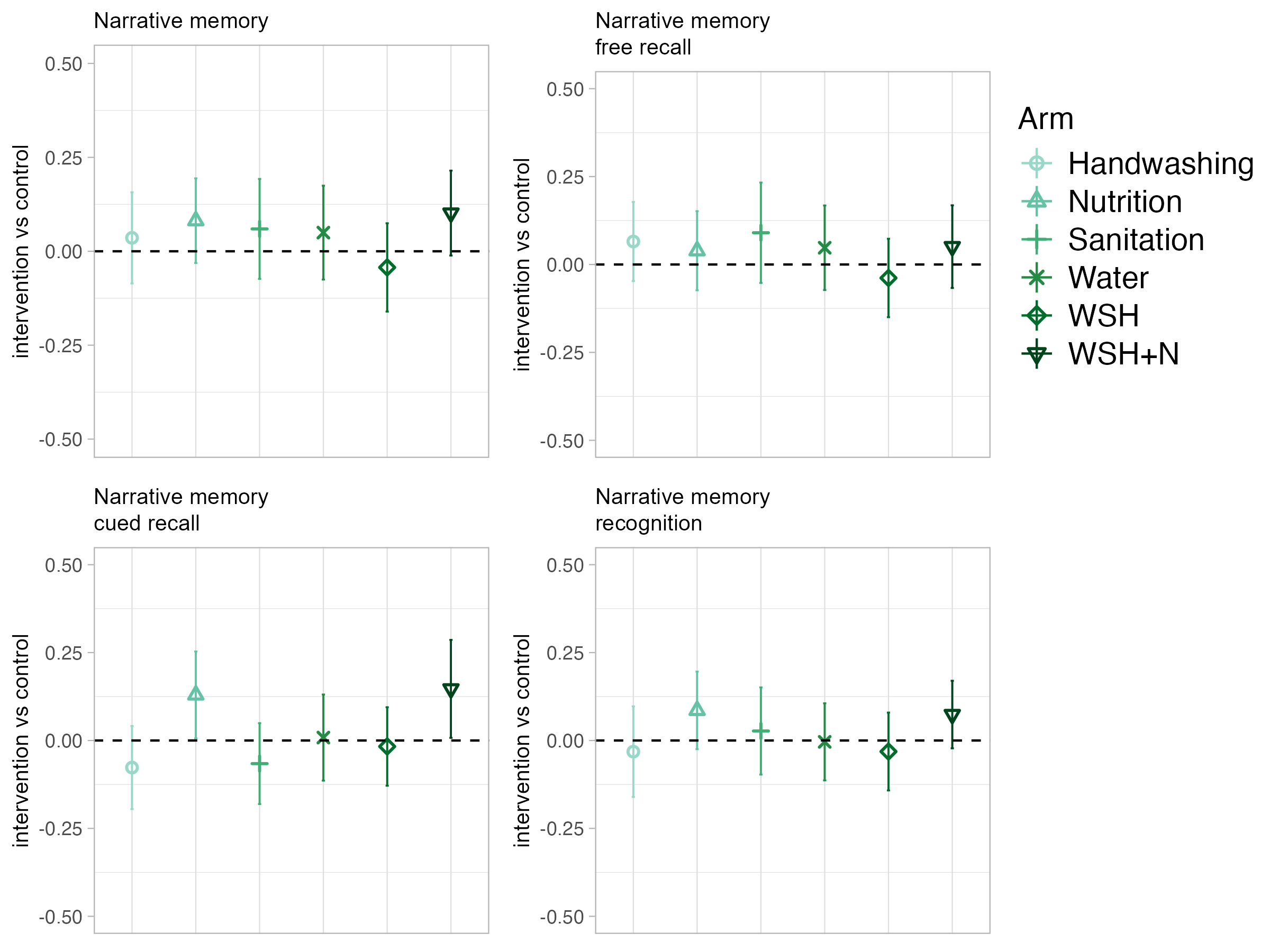


Point estimates reflect the mean difference between each intervention arm and the control arm from generalized linear models that adjust for child age, measurement period (pre-COVID or during COVID), and prognostic baseline control variables (significant at p<0.20 in a likelihood ratio test) to increase precision (for example of included control variables see Table G). All outcomes are presented as internally standardized z-scores, and all results account for the clustered study design through Huber-White robust standard errors clustered at the block-level. Bars represent 95% confidence intervals.

**Figure C. Subgroup analysis for SDQ prosocial outcome**

**
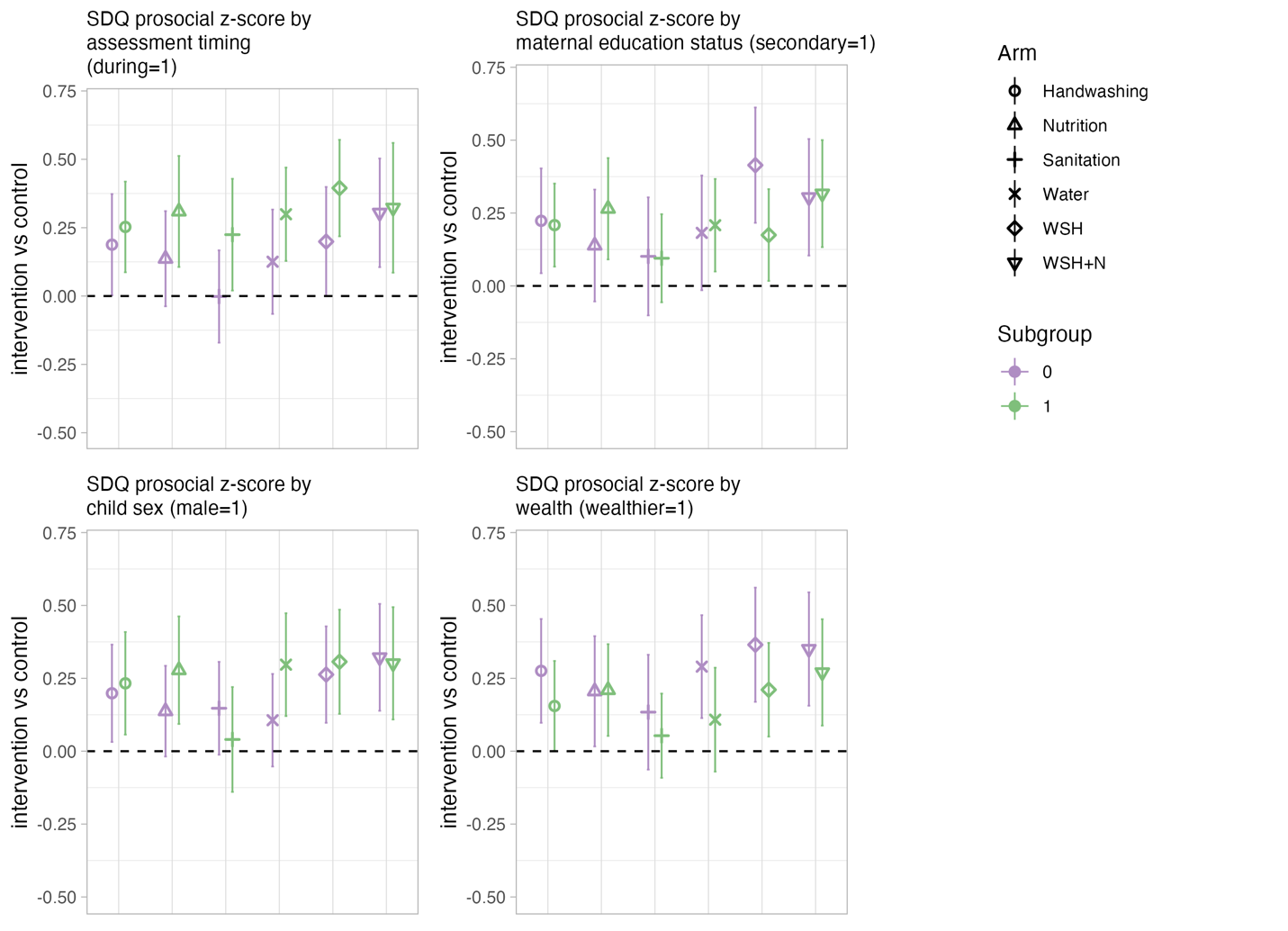
**

Point estimates reflect the mean difference between each intervention arm and the control arm in each subgroup, from generalized linear models that adjust for child age, measurement period (pre-COVID or during COVID), and prognostic baseline control variables (significant at p<0.20 in a likelihood ratio test) to increase precision (for example of included control variables see Table G). All outcomes are presented as internally standardized z-scores, and all results account for the clustered study design through Huber-White robust standard errors clustered at the block-level. Bars represent 95% confidence intervals.

**Figure D. Subgroup analysis for SDQ difficulties**


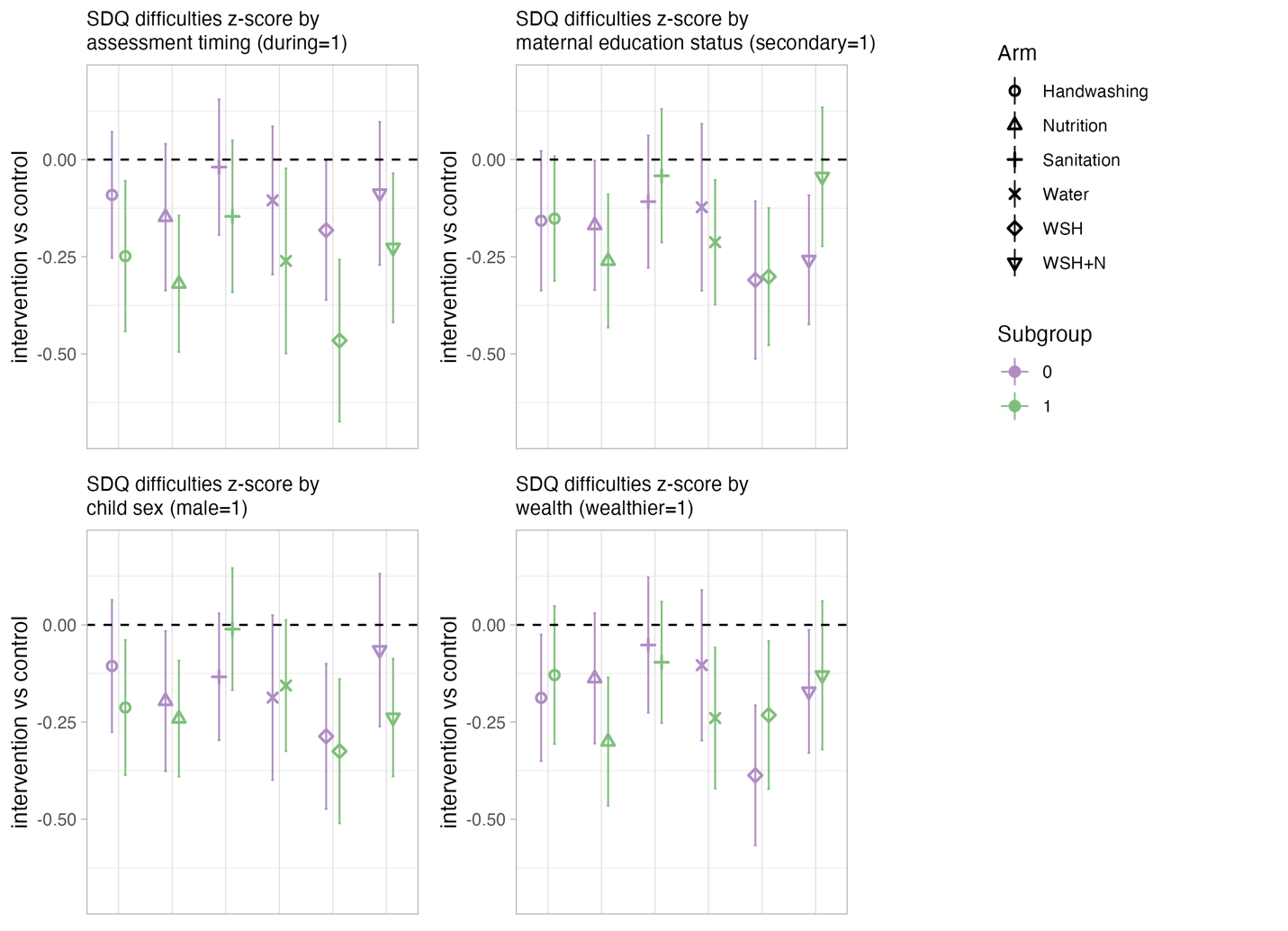
Point estimates reflect the mean difference between each intervention arm and the control arm in each subgroup, from generalized linear models that adjust for child age, measurement period (pre-COVID or during COVID), and prognostic baseline control variables (significant at p<0.20 in a likelihood ratio test) to increase precision (for example of included control variables see Table G). All outcomes are presented as internally standardized z-scores, and all results account for the clustered study design through Huber-White robust standard errors clustered at the block-level. Bars represent 95% confidence intervals.

**Figure E. Subgroup analysis for Fine motor outcome**


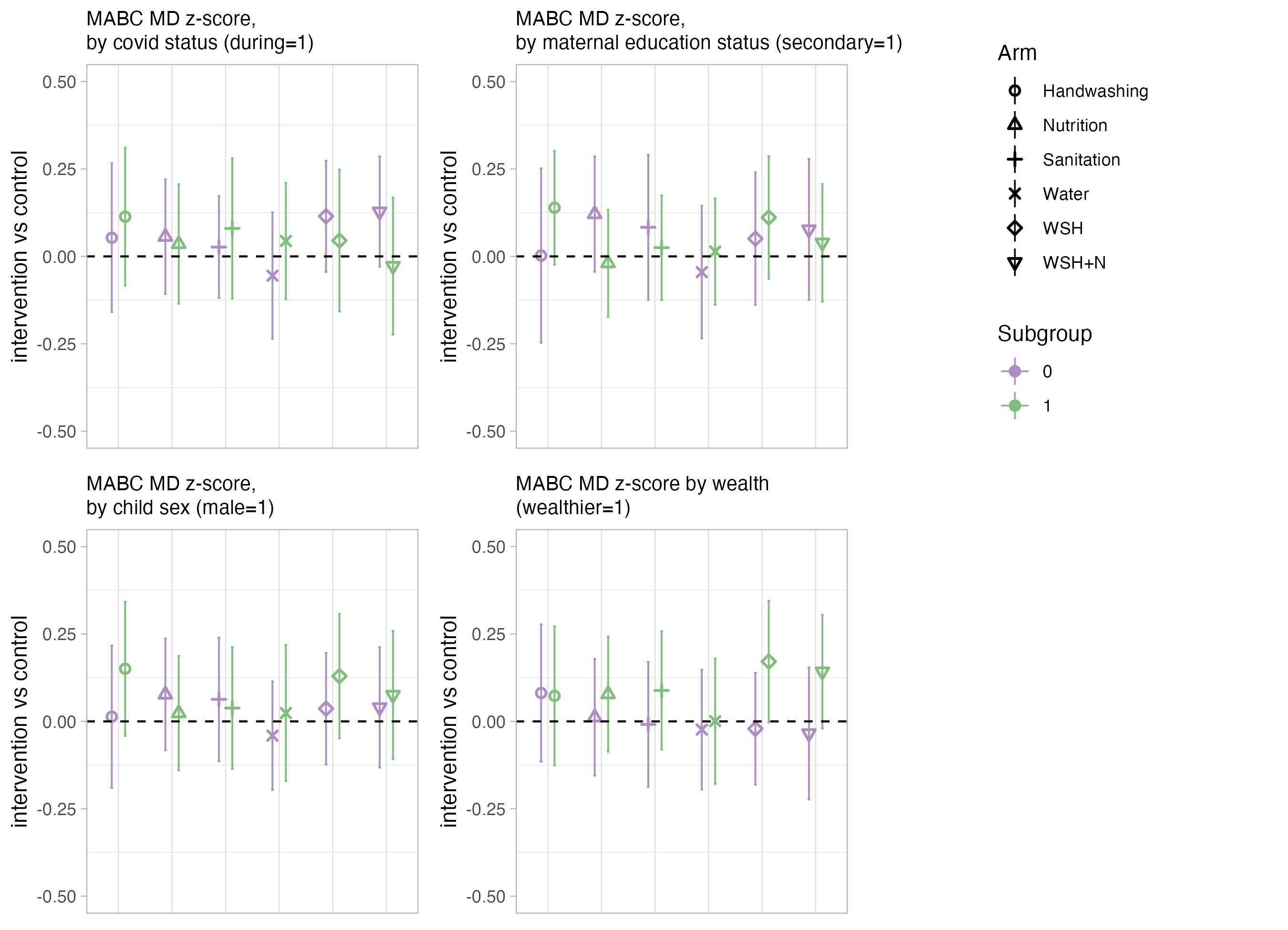


Point estimates reflect the mean difference between each intervention arm and the control arm in each subgroup, from generalized linear models that adjust for child age, measurement period (pre-COVID or during COVID), and prognostic baseline control variables (significant at p<0.20 in a likelihood ratio test) to increase precision (for example of included control variables see Table G). All outcomes are presented as internally standardized z-scores, and all results account for the clustered study design through Huber-White robust standard errors clustered at the block-level. Bars represent 95% confidence intervals.

**Figure F. Subgroup analysis for Math achievement**

**
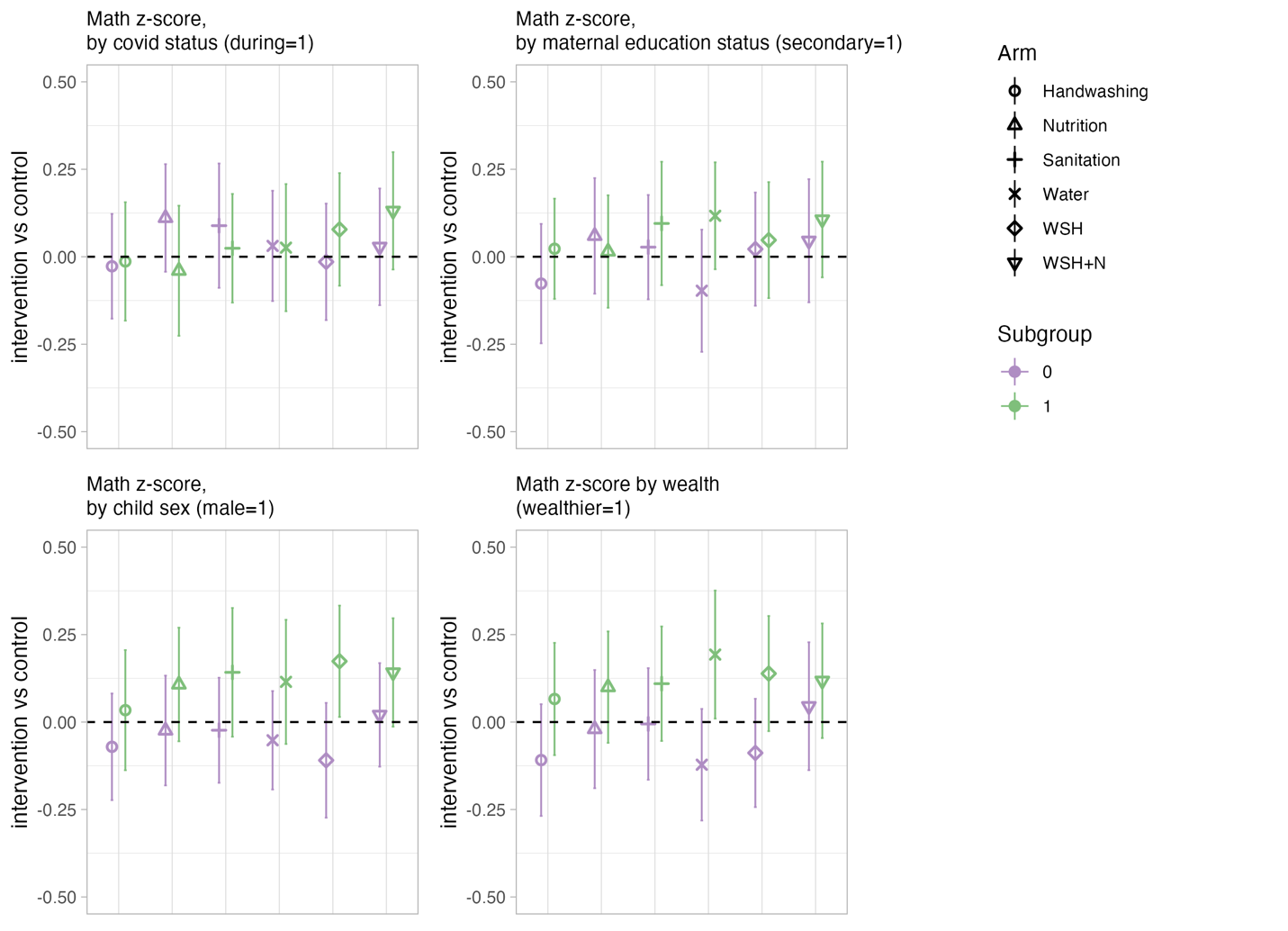
**

Point estimates reflect the mean difference between each intervention arm and the control arm in each subgroup, from generalized linear models that adjust for, measurement period (pre-COVID or during COVID), and prognostic baseline control variables (significant at p<0.20 in a likelihood ratio test) to increase precision (for example of included control variables see Table G). All outcomes are presented as internally standardized z-scores, and all results account for the clustered study design through Huber-White robust standard errors clustered at the block-level. Bars represent 95% confidence intervals.

**Figure G. Subgroup analysis for Reading achievement
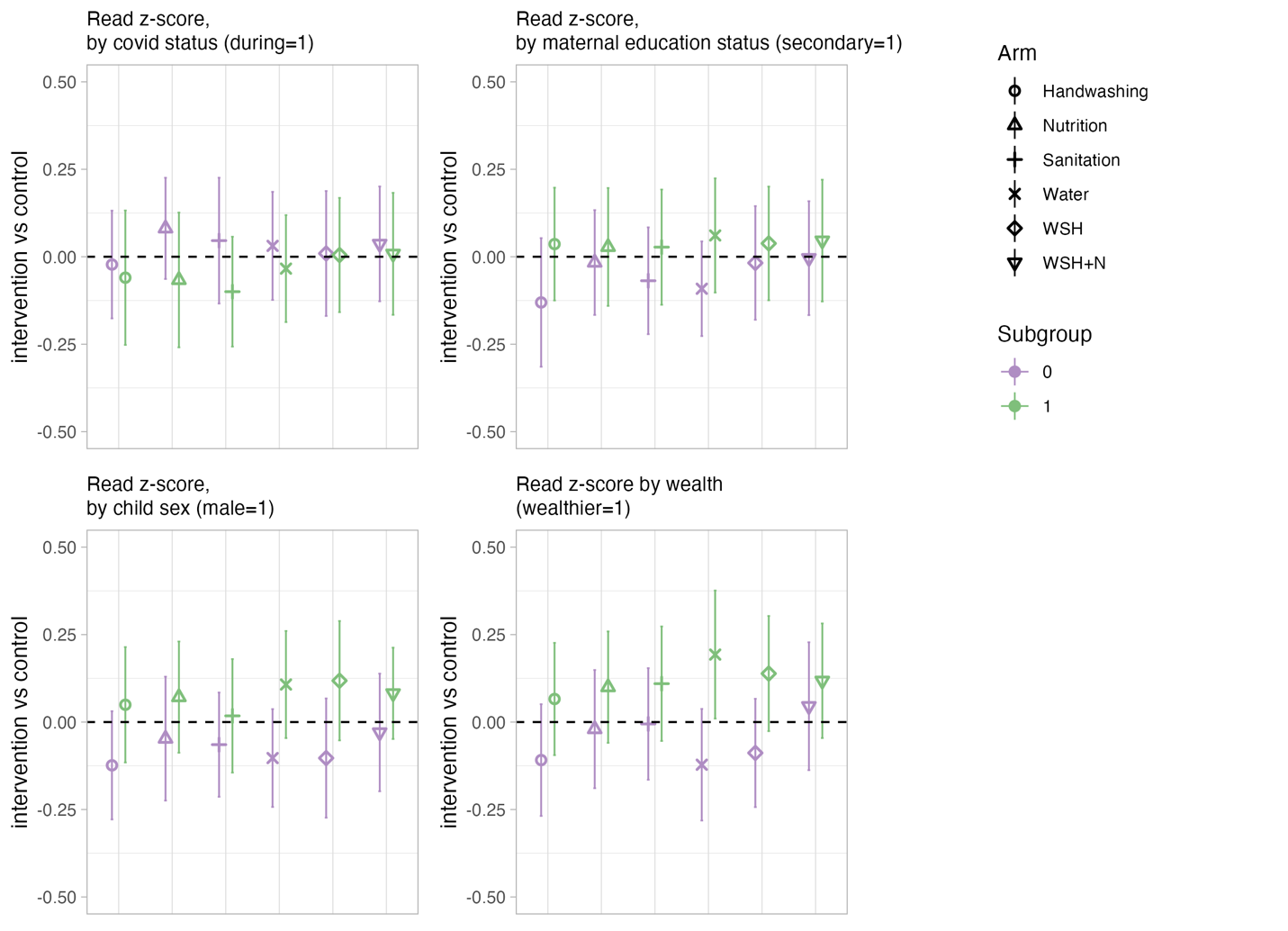
**

Point estimates reflect the mean difference between each intervention arm and the control arm in each subgroup, from generalized linear models that adjust for, measurement period (pre-COVID or during COVID), and prognostic baseline control variables (significant at p<0.20 in a likelihood ratio test) to increase precision (for example of included control variables see Table G). All outcomes are presented as internally standardized z-scores, and all results account for the clustered study design through Huber-White robust standard errors clustered at the block-level. Bars represent 95% confidence intervals.

**Figure H. Subgroup analysis for Spelling achievement
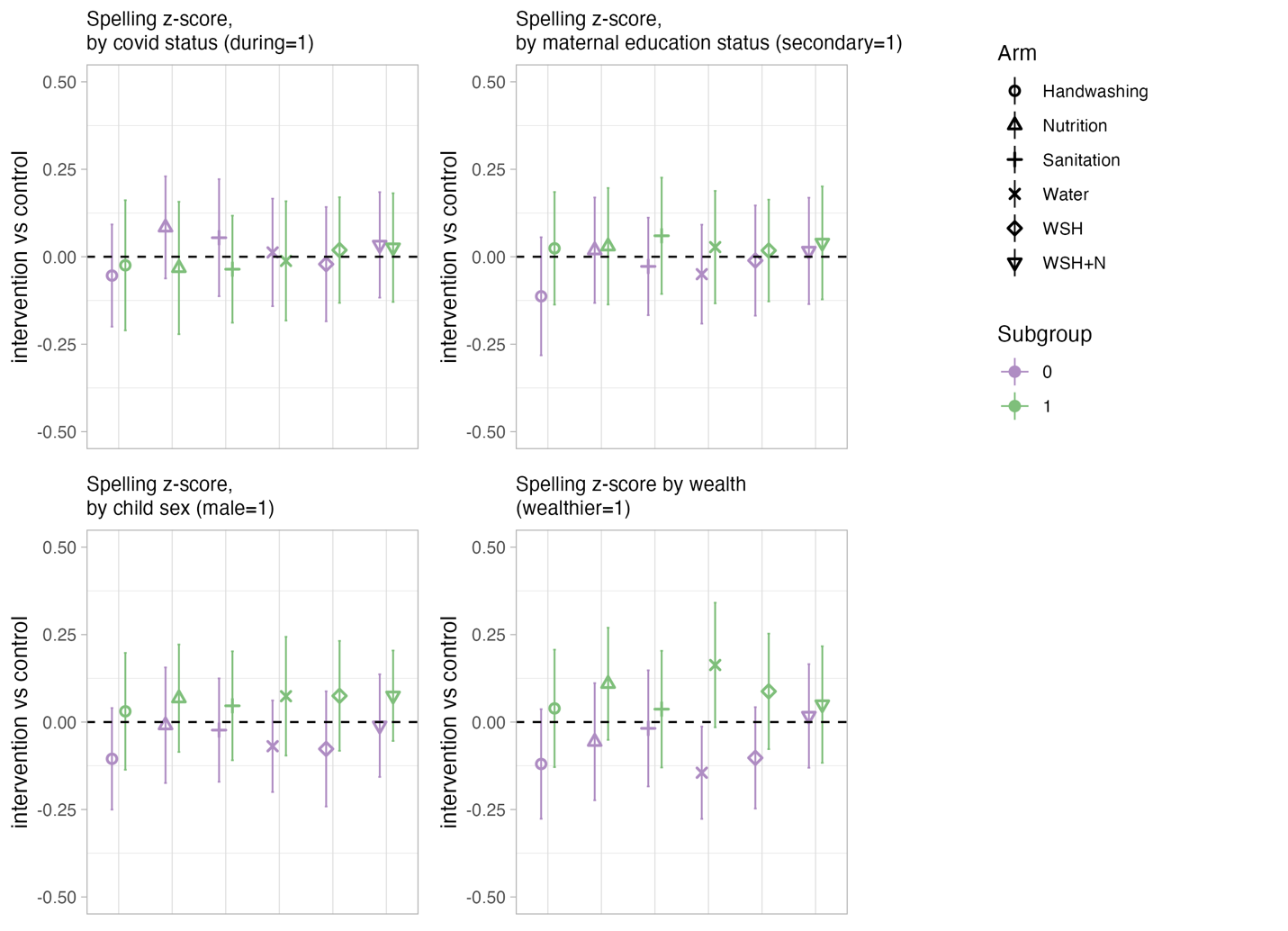
**

Point estimates reflect the mean difference between each intervention arm and the control arm in each subgroup, from generalized linear models that adjust for, measurement period (pre-COVID or during COVID), and prognostic baseline control variables (significant at p<0.20 in a likelihood ratio test) to increase precision (for example of included control variables see Table G). All outcomes are presented as internally standardized z-scores, and all results account for the clustered study design through Huber-White robust standard errors clustered at the block-level. Bars represent 95% confidence intervals.

**Figure I. Subgroup for Narrative memory**
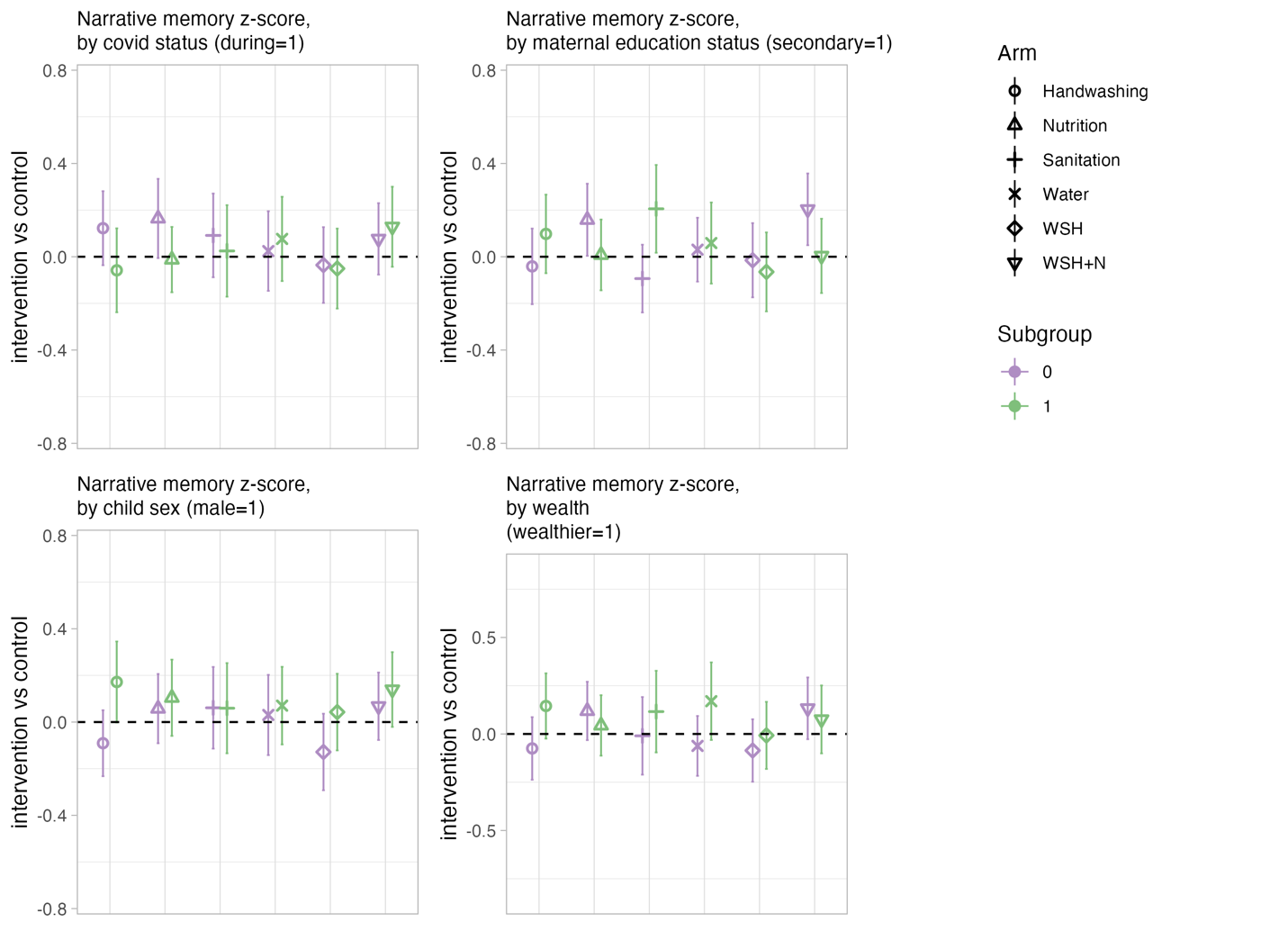


Point estimates reflect the mean difference between each intervention arm and the control arm in each subgroup, from generalized linear models that adjust for, measurement period (pre-COVID or during COVID), and prognostic baseline control variables (significant at p<0.20 in a likelihood ratio test) to increase precision (for example of included control variables see Table G). All outcomes are presented as internally standardized z-scores, and all results account for the clustered study design through Huber-White robust standard errors clustered at the block-level. Bars represent 95% confidence intervals.

**Figure J. Subgroup for Corsi blocks
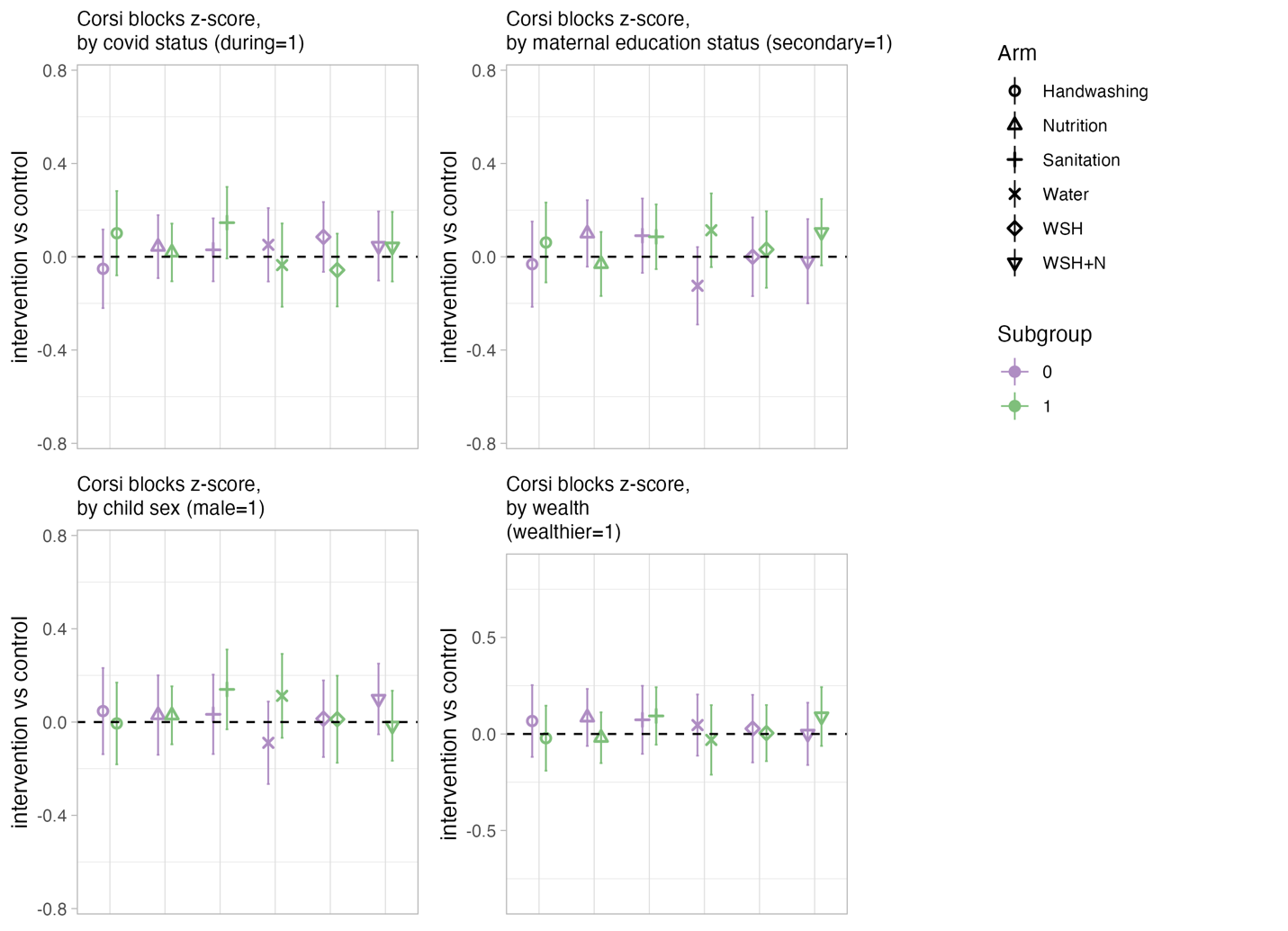
**

Point estimates reflect the mean difference between each intervention arm and the control arm in each subgroup, from generalized linear models that adjust for, measurement period (pre-COVID or during COVID), and prognostic baseline control variables (significant at p<0.20 in a likelihood ratio test) to increase precision (for example of included control variables see Table G). All outcomes are presented as internally standardized z-scores, and all results account for the clustered study design through Huber-White robust standard errors clustered at the block-level. Bars represent 95% confidence intervals.

**Figure K. Subgroup for Forward word span**
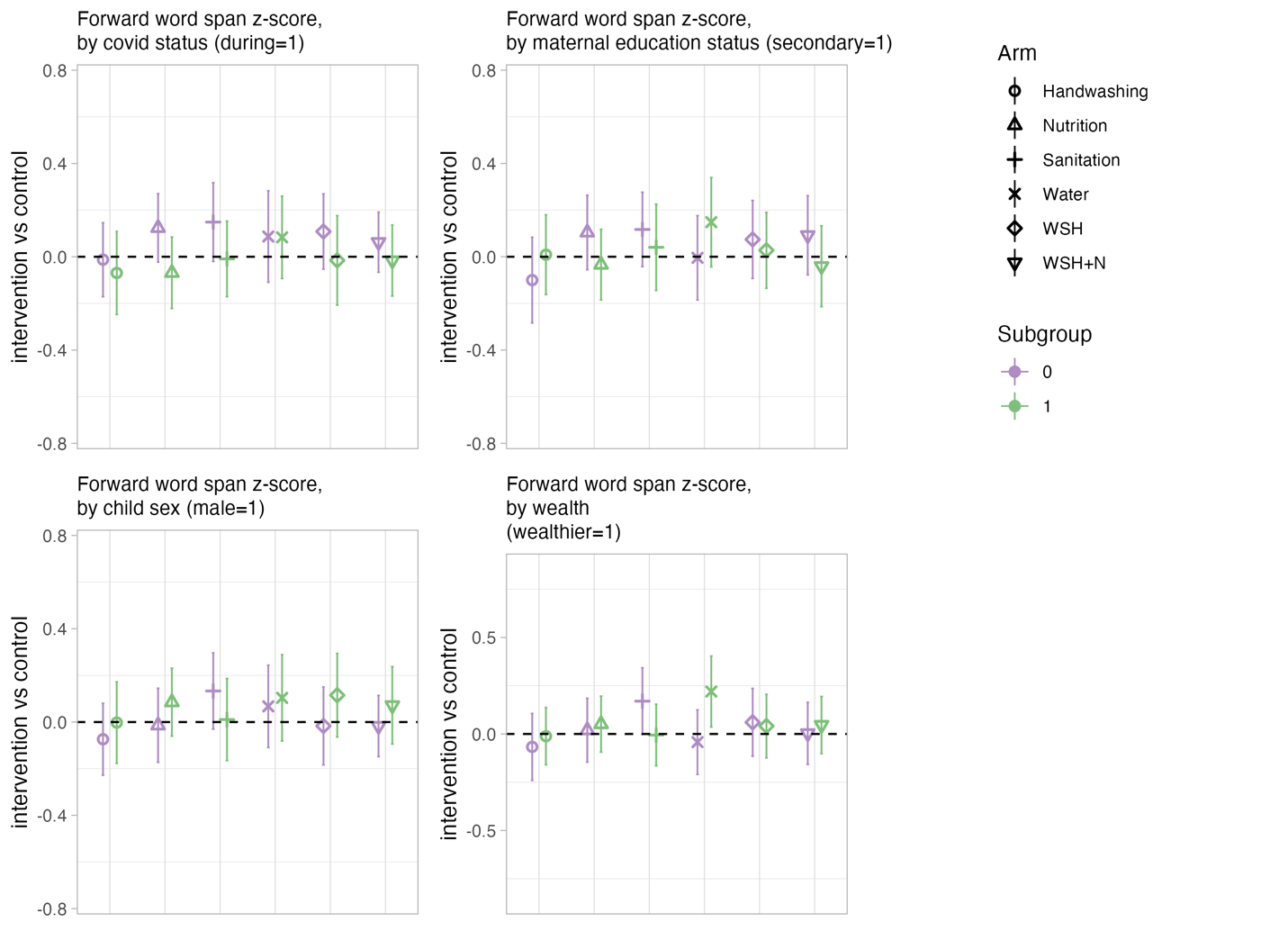


Point estimates reflect the mean difference between each intervention arm and the control arm in each subgroup, from generalized linear models that adjust for, measurement period (pre-COVID or during COVID), and prognostic baseline control variables (significant at p<0.20 in a likelihood ratio test) to increase precision (for example of included control variables see Table G). All outcomes are presented as internally standardized z-scores, and all results account for the clustered study design through Huber-White robust standard errors clustered at the block-level. Bars represent 95% confidence intervals.

**Figure L. Subgroup analysis for HOME
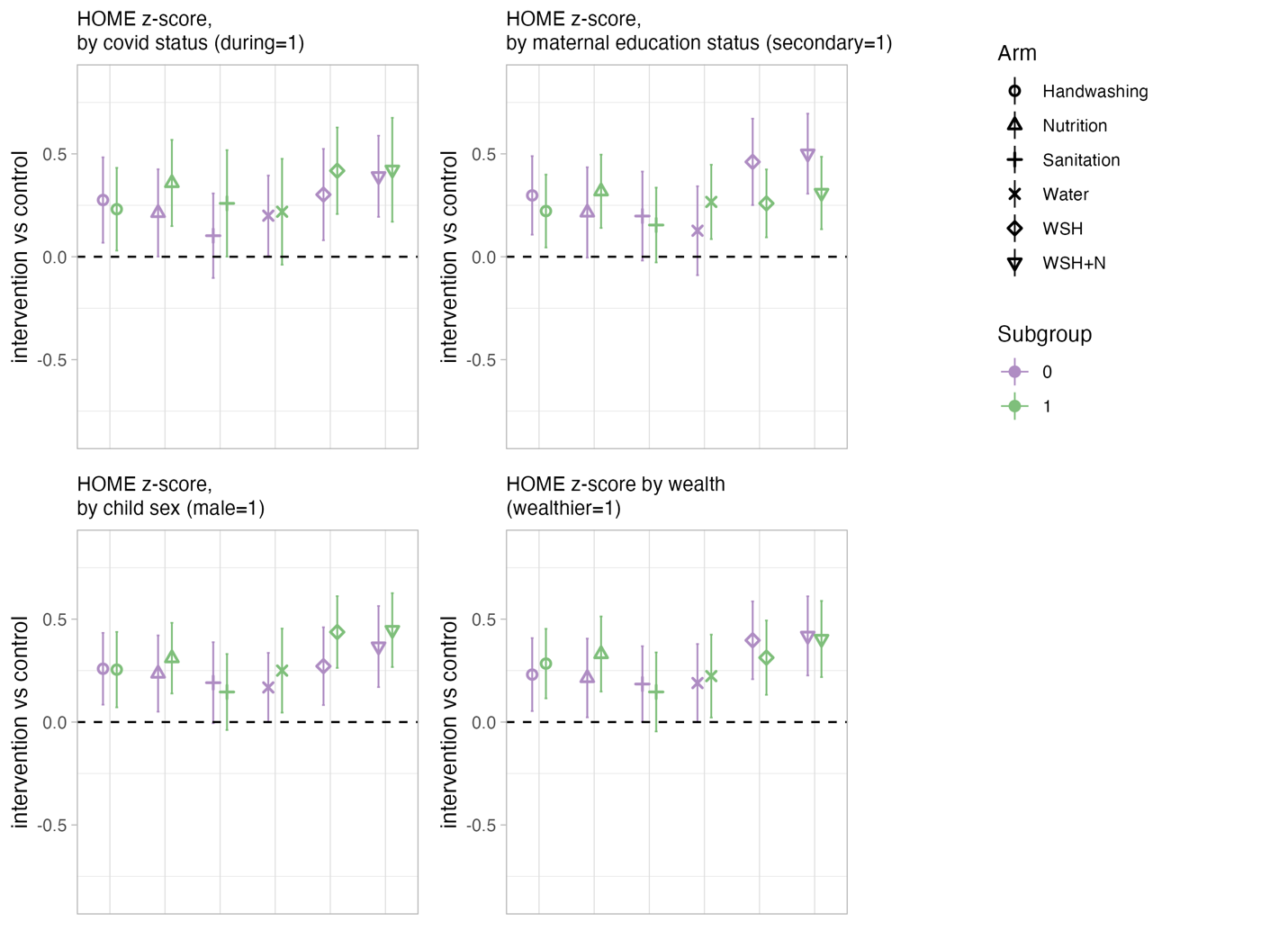
**

Point estimates reflect the mean difference between each intervention arm and the control arm in each subgroup, from generalized linear models that adjust for, measurement period (pre-COVID or during COVID), and prognostic baseline control variables (significant at p<0.20 in a likelihood ratio test) to increase precision (for example of included control variables see Table G). All outcomes are presented as internally standardized z-scores, and all results account for the clustered study design through Huber-White robust standard errors clustered at the block-level. Bars represent 95% confidence intervals.

**Figure M. Subgroup analysis for Maternal Depressive symptoms**
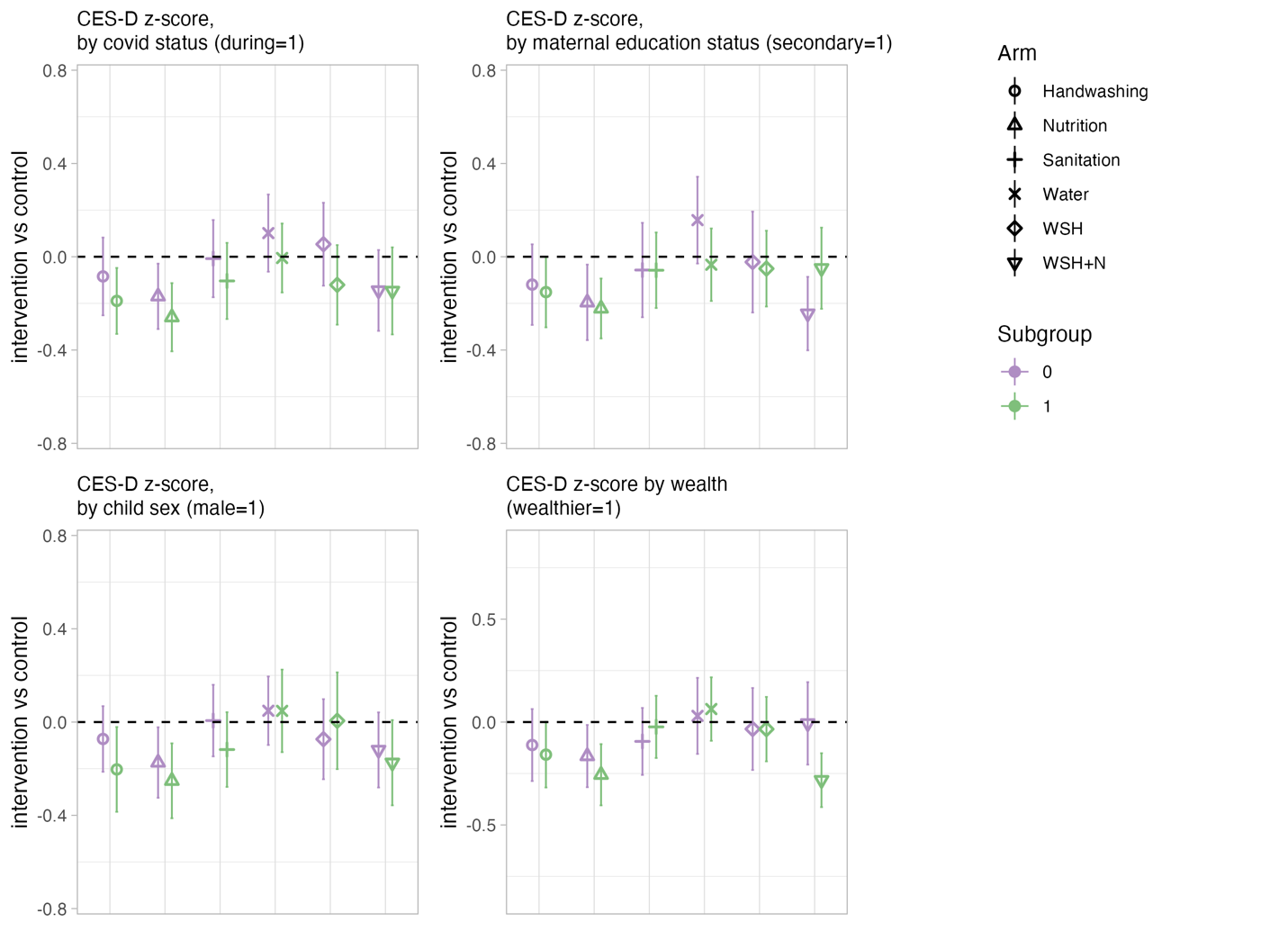


Point estimates reflect the mean difference between each intervention arm and the control arm in each subgroup, from generalized linear models that adjust for, measurement period (pre-COVID or during COVID), and prognostic baseline control variables (significant at p<0.20 in a likelihood ratio test) to increase precision (for example of included control variables see Table G). All outcomes are presented as internally standardized z-scores, and all results account for the clustered study design through Huber-White robust standard errors clustered at the block-level. Bars represent 95% confidence intervals. Depressive symptoms are maternal depressive symptoms measured by the Center for Epidemiologic Studies 20-question depression measure (CES-D)

**References**

1. Luby SP, Rahman M, Arnold BF, Unicomb L, Ashraf S, Winch PJ, et al. Effects of water quality, sanitation, handwashing, and nutritional interventions on diarrhoea and child growth in rural Bangladesh: a cluster randomised controlled trial. The Lancet Global Health. 2018;6: e302–e315. doi:10.1016/S2214-109X(17)30490-4
