# Supplementary material for: Effects of early water, sanitation, handwashing, and nutrition interventions on child development at school age: a follow-on study of a cluster-randomized trial in rural Bangladesh
Source: PLoS Med. 2025 Dec 16;22(12):e1004793. doi: 10.1371/journal.pmed.1004793 (PMC12707674; doi:10.1371/journal.pmed.1004793)
Supplement: S2 Checklist — Completed CONSERVE-CONSORT checklist. This file reports the changes made to the study as a result of the COVID-19 pandemic as per the CONSERVE-CONSORT checklist. (DOCX) [file pmed.1004793.s003.docx]

**CONSERVE Checklist**

| CONSERVE-CONSORT Extension: | | | | | |
| --- | --- | --- | --- | --- | --- |
| Item | Item Title | Description | | | Page No. |
| I. | Extenuating Circumstances | Describe the circumstances and how they constitute extenuating circumstances. | | | Results Paragraph 2 |
| II. | Important Modifications | 1. Describe how the modifications are important modifications. | | | Results Paragraph 2 |
|  |  | 1. Describe the impacts and mitigating strategies, including their rationale and implications for the trial. | | | Results Paragraph 2 |
|  |  | 1. Provide a modification timeline. | | | Results Paragraph 2 |
| III. | Responsible Parties | State who planned, reviewed and approved the modifications. | | | NA |
| IV. | Interim data | If modifications were informed by trial data, describe how the interim data were used, including whether they were examined by study group, and whether the individuals reviewing the data were blinded to the treatment allocation. | | | NA |
| CONSORT Number and Item | | For each row, if important modifications occurred check “direct impact” and/or “mitigating strategy” and describe the changes in the trial manuscript or supplement. Check “no change” for items that are unaffected in the extenuating circumstance. | | | Page No. |
|  |  | No Change | Impact* | Mitigating Strategy** |  |
| 1 | Title and abstract | X |  |  |  |
| 2 | Introduction | X |  |  |  |
| 3 | Methods: Trial Design | X |  |  |  |
| 4 | Methods: Participants | X |  |  |  |
| 5 | Methods: Interventions | X |  |  |  |
| 6 | Methods: Outcomes | X |  |  |  |
| 7 | Methods: Sample Size | X |  |  |  |
| 8-10 | Methods: Randomisation | X |  |  |  |
| 11 | Methods: Blinding | X |  |  |  |
| 12 | Methods: Statistical methods |  |  | X | Statistical analysis |
| 13 | Results: Participant flow | X |  |  |  |
| 14 | Results: Recruitment | X |  |  |  |
| 15 | Results: Baseline data | X |  |  |  |
| 16 | Results: Numbers analysed | X |  |  |  |
| 17 | Results: Outcomes and estimation |  |  | X | Statistical analysis |
| 18 | Results: Ancillary analyses |  |  |  |  |
| 19 | Results: Harms | X |  |  |  |
| 20 | Discussion: Limitations |  |  | X | Discussion 2^nd^ last paragraph |
| 21 | Discussion: Generalisability | X |  |  |  |
| 23 | Other information: Registration | X |  |  |  |
| 24 | Other information: Protocol | X |  |  |  |
| 25 | Other information: Funding | X |  |  |  |
| *Aspects of the trial that are directly affected or changed by the extenuating circumstance and are not under the control of investigators, sponsor or funder.  **Aspects of the trial that are modified by the study investigators, sponsor or funder to respond to the extenuating circumstance or manage the direct impacts on the trial. | | | | | |

Citation: Orkin AM, Gill PJ, Ghersi D, Campbell L, Sugarman J, Emsley R, Steg PG, Weijer C, Simes J, Rombey T, Williams HC, Wittes J, Moher D, Richards DP, Kasamon Y, Getz K, Hopewell S, Dickersin K, Wu T, Ayala AP, Schulz KF, Calleja S, Boutron I, Ross JS, Golub RM, Khan KM, Mulrow C, Siegfried N, Heber J, Lee N, Kearney PR, Wanyenze RK, Hróbjartsson A, Williams R, Bhandari N, Jüni P, Chan AW; CONSERVE Group. Guidelines for Reporting Trial Protocols and Completed Trials Modified Due to the COVID-19 Pandemic and Other Extenuating Circumstances: The CONSERVE 2021 Statement. JAMA. 2021.
