# Supplementary material for: Effects of early water, sanitation, handwashing, and nutrition interventions on child development at school age: a follow-on study of a cluster-randomized trial in rural Bangladesh
Source: PLoS Med. 2025 Dec 16;22(12):e1004793. doi: 10.1371/journal.pmed.1004793 (PMC12707674; doi:10.1371/journal.pmed.1004793)
Supplement: S1 File — (PDF) [file pmed.1004793.s004.pdf]

WASH Benefits Bangladesh follow up: Child development outcomes analysis plan

Authors of statistical analysis plan: Fahmida Tofail, Helen O. Pitchik, Abul K.M. Shoab, Lia C. H. Fernald, Steve Luby, Peter Winch, Sheuli Islam, Rizwana Khan, Mahbub Rahman

With input from:

1. Scientific background and rationale

Children growing up in poverty are exposed to multiple psychological, physiological and environmental risk factors that shape their development.[1] Poverty increases exposure to poor sanitation and hygiene, acute and chronic infection, poor nutrition, food insecurity, abuse and neglect, and stress.[2] These conditions can have strong and enduring effects on child development across many domains.[3] Globally, millions of children experience delays in physical health and cognitive development, due to their exposures to poverty and related issues.[1] In low-and middle-income countries (LMICs), children experience a disproportionally high burden of exposure to poverty and related risk factors for delayed development including of poor health and nutrition, inadequate responsive caregiving, and a lack of opportunities for early learning. [1]

Water, sanitation and hygiene (WASH) interventions have the potential to positively affect the developmental trajectories of children by reducing enteric pathogen infection, improving child health, and altering parental care practices.[4] A recent cluster-randomized controlled trial (cRCT) in Bangladesh (WASH-Benefits, or WASH-B, ClinicalTrials.gov Identifier: NCT01590095),[5] found that improvements in WASH or nutrition supported by intensive interpersonal communication, when delivered either individually or in combination, contributed to improvements in child development outcomes at 1 and 2 years of age.[6] Mothers in all intervention groups reported lower depressive symptoms than mothers in the control households.

We have followed-up these children and assessed them at 6-8 years old, and propose to examine whether the improvements in cognition among children and the maternal mental health findings are sustained in middle childhood.

2. Study hypotheses, exposures, and outcomes

<u>Hypothesis:</u> Early intervention effects on child development outcomes will persist to middle childhood.

<u>Exposures:</u> Wash, Sanitation, Hygiene, W+S+H, Nutrition and W+S+H+N intervention arms each individually vs control. Additionally, we will compare W+S+H+N vs. Nutrition, and W+S+H+N vs W+S+H to isolate the additive effects of W+S+H and N, respectively.

<u>Outcomes:</u>

<u>Primary:</u>

Wechsler Pre & Primary School Intelligence (WPPSI-IV):

- Full Scale IQ (FSIQ)

- 3 Primary Index Scores (Verbal Comprehension Index, Fluid Reasoning Index, Working Memory Index)
- 1 measure of processing speed (bug search and animal coding)
- 3 ancillary index scores: General Ability Index (GAI), Nonverbal Index (NVI), and Cognitive Proficiency Index (CPI)

Fine Motor Abilities (based on the Movement Assessment Battery for Children assessment): Three measures of fine motor ability: Posting Coins/Placing Pegs, Threading Lace, and Drawing Trails Scores, and a combined Manual Dexterity

Strengths and difficulties questionnaire: Prosocial behaviors (5 items sum score), Difficult behaviors (20 items sum score)

Executive functioning: Digit span, Corsi-block, and narrative memory

School achievement test: Reading, Literacy and Numeracy scores following the principles of wide range achievement test

<u>Secondary:</u>
Maternal Depression: Depression score using 20 questions of Centre for Epidemiologic Studies Depression (CES-D) Scale

Home Environment: Middle childhood Home Observation Measurement of the Environment (HOME)

Standardization: All child-development outcomes will be converted to z-scores for comparability across tests, where the scores for each child are standardized to the control group mean and standard deviation. Each outcome will be tested for trends by age, and if they exist we will conduct age-standardization using local-mean standardization with 4 month age bands. Children with scores that are 4 or more standard deviations from the mean on each outcome will be excluded from that specific analyses. Similarly children who were over 7 years and 7 months at the time of WPPSI test, will be excluded from the analysis where WPPSI composite scores will be used as they are above the age-range cutoff. Supplementary analyses will be done with raw scores for each subscale, and scaled scores for WPPPSI-IV.

Covariates: demographic and socioeconomic variables collected at baseline will be assessed for inclusion in each model. Covariates will be assessed by likelihood ratio test and those that are associated with $p<0.20$ will be included in the adjusted analysis. Potential covariates for inclusion will be: Child sex, maternal age, maternal height (in cm), parents' education in year of schooling, number of children younger than 18 years in household, total number of people in the compound, food insecurity of household (measured using Household Hunger Scale), housing materials (construction materials and utilities), household assets, distance to water source, and month of measurement. Covariates that have low prevalence (<10%) or are highly

correlated (>0.70) will be excluded. Time of assessment (pre-COVID or during COVID will be controlled for in all analyses. We will control for concurrent child age in all models.

3. Analysis

All analyses will be intention to treat. Randomization was geographically pair-matched in blocks of eight clusters, so unadjusted mean differences will be determined using generalized linear models accounting for pair matching and block-level clustering. For each outcome we will present unadjusted and adjusted comparisons between each arm and the control arm as well as comparisons between W+S+H+N vs. Nutrition, and W+S+H+N vs W+S+H to isolate the additive effects of W+S+H and N, respectively.

Sensitivity analysis:
We will compare baseline covariates of participants who were followed-up, and those who were lost at the 5-year follow-up assessment. If there are differences, we will conduct a supplementary analysis using inverse probability weighted-intention to treat analysis.

We will conduct subgroup analysis by the following variables:
a) maternal education at baseline
b) child age assessment
c) child sex at birth
d) measurement before or during the COVID-19 pandemic
e) socioeconomic status at baseline
f) closer vs further from Dhaka

References

1 Black MM, Walker SP, Fernald LCH, *et al.* Early childhood development coming of age: science through the life course. *The Lancet* 2017;**389**:77–90. doi:10.1016/S0140-6736(16)31389-7

2 Britto PR, Lye SJ, Proulx K, *et al.* Nurturing care: promoting early childhood development. *The Lancet* 2017;**389**:91–102. doi:10.1016/S0140-6736(16)31390-3

3 Richter LM, Daelmans B, Lombardi J, *et al.* Investing in the foundation of sustainable development: pathways to scale up for early childhood development. *The Lancet* 2017;**389**:103–18. doi:10.1016/S0140-6736(16)31698-1

4 Oriá RB, Murray-Kolb LE, Scharf RJ, *et al.* Early-life enteric infections: relation between chronic systemic inflammation and poor cognition in children. *Nutr Rev* 2016;**74**:374–86. doi:10.1093/nutrit/nuw008

5 Arnold BF, Null C, Luby SP, *et al.* Cluster-randomised controlled trials of individual and combined water, sanitation, hygiene and nutritional interventions in rural Bangladesh and Kenya: the WASH Benefits study design and rationale. *BMJ Open* 2013;**3**:e003476. doi:10.1136/bmjopen-2013-003476

6 Tofail F, Fernald LC, Das KK, *et al.* Effect of water quality, sanitation, hand washing, and nutritional interventions on child development in rural Bangladesh (WASH Benefits Bangladesh): a cluster-randomised controlled trial. *Lancet Child Adolesc Health* 2018;**2**:255–68. doi:10.1016/S2352-4642(18)30031-2
